# Investigation of the acetic acid stress response in *Saccharomyces cerevisiae* with mutated H3 residues

**DOI:** 10.15698/mic2023.10.806

**Published:** 2023-08-18

**Authors:** Nitu Saha, Swati Swagatika, Raghuvir Singh Tomar

**Affiliations:** 1 Laboratory of Chromatin Biology, Department of Biological Sciences, Indian Institute of Science Education and Research Bhopal, 462066, Madhya Pradesh, India.

**Keywords:** acetic acid, H3 point mutants, H3 N-terminal tail truncation mutants, AIF1, reactive oxygen species

## Abstract

Enhanced levels of acetic acid reduce the activity of yeast strains employed for industrial fermentation-based applications. Therefore, unraveling the genetic factors underlying the regulation of the tolerance and sensitivity of yeast towards acetic acid is imperative for optimising various industrial processes. In this communication, we have attempted to decipher the acetic acid stress response of the previously reported acetic acid-sensitive histone mutants. Revalidation using spot-test assays and growth curves revealed that five of these mutants, viz., H3K18Q, H3S28A, H3K42Q, H3Q68A, and H3F104A, are most sensitive towards the tested acetic acid concentrations. These mutants demonstrated enhanced acetic acid stress response as evidenced by the increased expression levels of *AIF1*, reactive oxygen species (ROS) generation, chromatin fragmentation, and aggregated actin cytoskeleton. Additionally, the mutants exhibited active cell wall damage response upon acetic acid treatment, as demonstrated by increased Slt2-phosphorylation and expression of cell wall integrity genes. Interestingly, the mutants demonstrated increased sensitivity to cell wall stress-causing agents. Finally, screening of histone H3 N-terminal tail truncation mutants revealed that the tail truncations exhibit general sensitivity to acetic acid stress. Some of these N-terminal tail truncation mutants viz., H3 [del 1-24], H3 [del 1-28], H3 [del 9-24], and H3 [del 25-36] are also sensitive to cell wall stress agents such as Congo red and caffeine suggesting that their enhanced acetic acid sensitivity may be due to cell wall stress induced by acetic acid.

## INTRODUCTION

Acetic acid, produced during alcoholic fermentation by *Saccharomyces cerevisiae*, is a stress and death-inducing agent [1]. Enhanced acetic acid levels are detrimental to yeast-based industrial fermentation processes such as wine production [1] and the production of bioethanol from lignocellulosic material [1, 2]. Thus, due to its production in widespread yeast-related industrial processes, the acetic acid study has always garnered particular importance [1]. Therefore, studies focused on elucidating the molecular basis of acetic acid production in yeast cultures and the mechanisms underlying acetic acid-mediated yeast cytotoxicity are being actively pursued [2]. Yeast cells exhibit hallmarks of mammalian cellular apoptosis, including DNA fragmentation, phosphatidylserine (PS) externalization, ROS production, and chromatin condensation [3, 4]. Pioneering work on a mutant of the cell division cycle gene, *CDC48,* in yeast, revealed molecular and morphological features specific to apoptosis in metazoan cells [3]. Subsequently, it was shown that several factors can trigger apoptotic features in yeast, including depletion of glutathione or treatment with hydrogen peroxide, which increases cellular oxygen radical levels [5]. Moreover, decreasing the ROS levels in the cells or maintaining hypoxia helped the yeast survive under both conditions [5] suggesting that the generation of ROS leads to apoptosis in the yeast cells. As in mammalian cells, yeast metacaspase Yca1 plays an important role in Regulated Cell Death (RCD) mediated by oxidative stress [6]. Similarities between apoptosis mechanisms in mammalian and yeast cells suggest that studying the mechanisms and genetic basis of apoptosis in yeast can provide valuable insights into the corresponding mammalian mechanisms as well, rendering such studies extremely important. Multiple studies have shown the concentration-dependent ability of weak acids to inhibit microbial growth [7] and that acetic acid can induce regulated cell death in *S. cerevisiae* [8]. One of the pathways for acetic acid-dependent RCD (AA-RCD) involves the release of cytochrome c from mitochondria that triggers a decrease in mitochondrial membrane potential leading to the RCD. AA-RCD also proceeds via a cytochrome c release-independent pathway that depends on the yeast caspase Yca1 protein that promotes oxidative stress-mediated apoptosis in yeast cells [6]. Yeast cells which undergo RCD tend to have a shorter life span, they accumulate ROS and show increased PS externalization with ageing [9]. Together, these processes lead to an enhanced death of cells compared to their wild-type (WT) counterparts [9].

Cell wall integrity (CWI) pathway gets triggered due to environmental challenges or cell wall stress. The pathway commences with the firing of signal from the sensors (Wsc1, 2 or 3, Mtl1 or Mid2) present at the cell surface which is coupled to Rho1, a G protein and the master regulator of the cell wall integrity pathway [10, 11]. Rho1 generates a diverse output through a cascade of molecules. The level of transcriptional output depends on protein kinase C (Pkc1), which Rho1 activates. Pkc1 phosphorylates the first protein of the MAPK cascade, MAPKKK (Mitogen-Activated Protein Kinase Kinase Kinase), which phosphorylates MAPKK (Mitogen-Activated Protein Kinase Kinase). MAPKK, in turn, phosphorylates the tyrosine and threonine residues on the activation loop of MAPK (Mitogen-Activated Protein Kinase) [12].

The histone mutant library was created by Dai *et al.*, 2008 to probe into the function of the nucleosome. It consists of histone variants containing substitutions of one or multiple amino acid residues or deletion of an amino acid stretch in the H3 or H4 core histones [13, 14]. To understand the acetic acid tolerance mechanism in *S. cerevisiae*, Liu *et al.*, 2014 screened the H3/H4 library and found that many point mutants were either sensitive or resistant to acetic acid stress [14]. The resistant strains displayed enhanced ethanol production capacity under acetic acid stress. Furthermore, the transcriptome profile of resistant strains revealed enhanced antioxidant gene expression [14]. The reported acetic acid-sensitive histone point mutants provided room for understanding the involvement of genetically diverse yeast strains in acetic acid sensitivity. We employed acetic acid-sensitive histone point mutant-expressing strains of this library to study the molecular basis of acetic acid sensitivity. Our studies revealed that five of the previously reported acetic acid-sensitive histone mutants viz., H3K18Q, H3S28A, H3K42Q, H3Q68A and H3F104A exhibit the hallmarks of molecular response to the stress and that the sensitive mutants show increased expression levels of *AIF1* gene. Deletion of *aif1* in WT, H3S28A and H3Q68A increases the survival of the cells against acetic acid insult. Additionally, the mutants demonstrate chromatin fragmentation, and enhanced ROS levels and marked differences in the actin distribution as compared to the WT cells. Furthermore, the mutants exhibit a shorter chronological life span (CLS) than the WT cells. We also found that the mutants show enhanced levels of Slt2-phosphorylation and sensitivity to cell wall perturbing agents. Notably, the mutant strains exhibit increased expression of *RLM1*, a cell wall integrity transcription factor and *PRM5*, one of the cell wall integrity pathway genes. The results indicate that these mutants exhibit increased cell wall stress in acetic acid. Thus, all these factors together can result in cell death of the acetic acid-sensitive mutants. We have also screened H3 N-terminal tail truncation mutants in acetic acid and found that almost all of them show the sensitive phenotype, out of which four deletions viz., H3[del 1-24], H3[del 1-28], H3[del 9-24] and H3[del 25-36] were the most sensitive. These mutants also showed sensitivity to cell wall perturbing agents. Understanding the basis of sensitivity in these tail truncation mutants would help provide better insight into the genetic basis of acetic acid sensitivity in the yeast system.

## RESULTS

### Revalidation of the previously identified acetic acid-sensitive histone H3 point mutants

**Figure 1:**
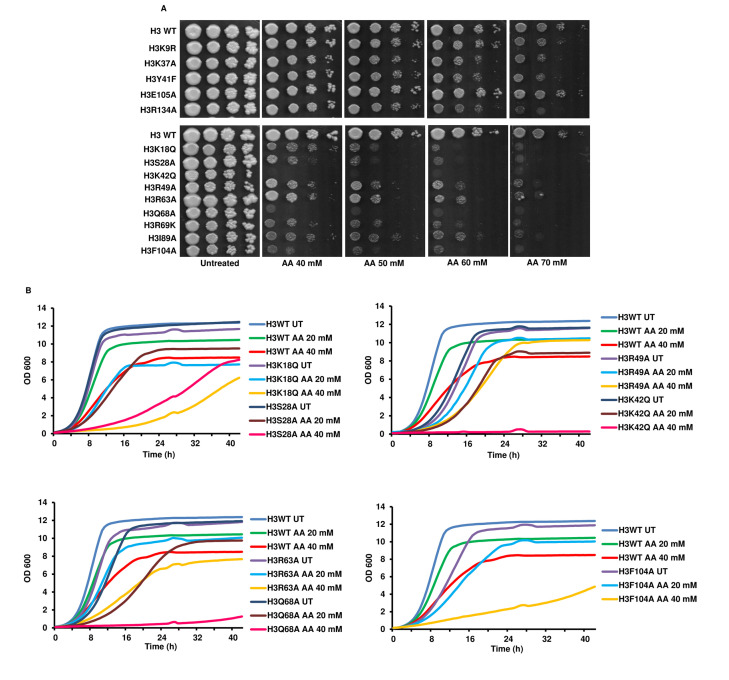
FIGURE 1: Revalidation of the previously identified acetic acid-sensitive histone H3 point mutants. **(A)** Spot test assay of the wild-type (H3 WT) and the mutants (at 10-fold dilution from left to right) in different concentrations of acetic acid (40, 50, 60 and 70 mM). The untreated plate represents YPD pH 4.5 without any acetic acid and shows the growth of cells at their full potential. Cells were allowed to grow at 30°C, and plates were scanned at 72 h. **(B)** Growth of the H3 WT and mutant cells in liquid culture under untreated (UT) or acetic acid treated (AA) (20, 40 mM) conditions. The graphs reflect the absorbance values at 600 nm at every 30' for the hours indicated in the x-axis.

We revalidated the previously identified acetic acid-sensitive mutants [14] by performing spot test assay with increasing concentrations of acetic acid (40, 50, 60 and 70 mM). As shown in **Fig. 1A**, many of the previously reported mutants exhibited sensitive phenotypes in the presence of acetic acid. Next, we checked the sensitivity of all the mutants in liquid culture by growing the cells until the exponential phase, and checking for their sensitivity towards the acetic acid measured through an increase in absorbance at 600 nm. We found that five mutants, namely, H3K18Q, H3S28A, H3K42Q, H3Q68A and H3F104A, exhibited higher sensitivity compared to WT cells at tested acetic acid 20 and 40 mM concentrations. Specifically, all five mutants showed marked differences in growth with the WT cells at a 40 mM concentration of acetic acid (**Fig. 1B**, Fig. S1)

### Acetic acid-sensitive mutants exhibit enhanced *AIF1* gene expression levels

**Figure 2:**
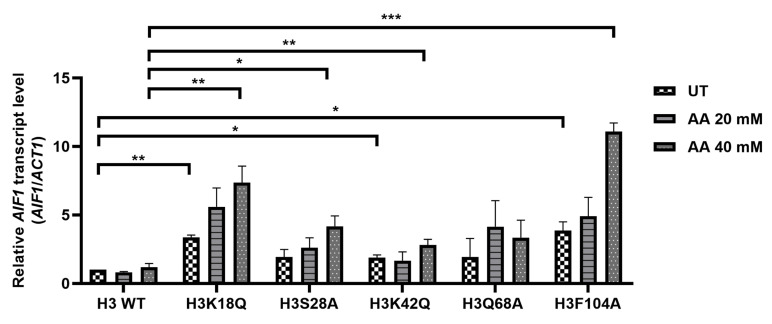
FIGURE 2: Acetic acid-sensitive mutants exhibit enhanced *AIF1* gene expression levels. *AIF1* mRNA level examined in the wild-type (H3 WT) and mutants under untreated (UT) or different concentrations of acetic acid (20-, 40-mM), the relative expression with respect to *ACT1* gene is shown. Statistical analysis was performed through unpaired Student's t-test with Welch's correction (GraphPad Prism 8 GraphPad Software, Inc). The mean ± SD of three biological replicates (n= 3) are shown. ***p < 0.001, **p (0.001 to 0.01), *p (0.01 to 0.05).

Acetic acid has been shown to induce yeast apoptosis [4]. We hypothesized that these acetic acid-hypersensitive histone mutants may have enhanced expression levels of the genes involved in apoptosis. To test this hypothesis, we checked the expression levels of genes related to yeast apoptosis. To this end, first, we determined the survival of WT and mutants in 20 or 40 mM acetic acid at different time points of 90' or 180' to confirm that both the WT and the mutant cells showed at least more than 50% survival at all the conditions (Fig. S2A, B, C and D). Acetic acid stress generates several responses in yeast. For example, acetic acid-mediated yeast apoptosis requires a yeast caspase like Yca1, which shows structural homology with the mammalian caspase. The features of yeast apoptosis, specifically the caspase activity, in the presence of apoptotic agents like hydrogen peroxide, were absent in the *yca1Δ* cells [6]. Nma111p, or nuclear mediator of apoptosis, interacts with the nuclear pore complex and exhibits a serine protease activity responsible for its pro-apoptotic function [15]. When not in mitochondria, Nuc1p triggers apoptotic cell death independent of the apoptosis-inducing factor or the metacaspases [16]. Aif1, a yeast homologue of mammalian AIF, relocates to the nucleus from the mitochondria under apoptotic stimulus. Overexpression of the Aif1 protein makes cells sensitive to acetic acid or hydrogen peroxide. *In vitro* experiments have shown that the protein degrades yeast nuclei and plasmid DNA, implicating its role in apoptosis through DNA fragmentation [17]. Thus, we checked the expression levels of *YCA1* [6], *NMA111* [15], *NUC1* [18] and *AIF1* [17] genes. We grew WT and mutant cells till the exponential phase, left the cells untreated or treated with different concentrations of acetic acid and prepared cDNA from the harvested cells. We then checked the expression of the genes involved in apoptosis and found that at acetic acid concentrations of 20 and 40 mM for 90' and 180', the mutants exhibited enhanced transcript levels of *AIF1* at 40 mM and 180' (Fig. S3A, B and C). In untreated conditions, the mutant H3K18Q showed almost 3.4-fold induction compared to the WT H3. Similarly, the mutants H3K42Q and H3F104A showed almost 1.9- or 3.9 times induction. Post-treatment with the acetic acid, while the WT did not exhibit significant induction of the *AIF1* gene, all the mutants H3K18Q, H3S28A, H3K42Q, H3F104A except H3Q68A showed significant inductions of the gene. H3K18Q, H3S28A, H3K42Q and H3F104A showed approximately 6.3-, 3.5-, 2.4- and 9.4-times induction, respectively, compared to the WT at 40 mM acetic acid concentration treatment for 3h (**Fig. 2**). On the contrary, other genes involved in yeast apoptosis did not show significant changes upon acetic acid treatment under the same conditions (Fig. S3A, B).

### Deleting *aif1* in the acetic acid-sensitive mutants increases their survival to different degrees in acetic acid

**Figure 3:**
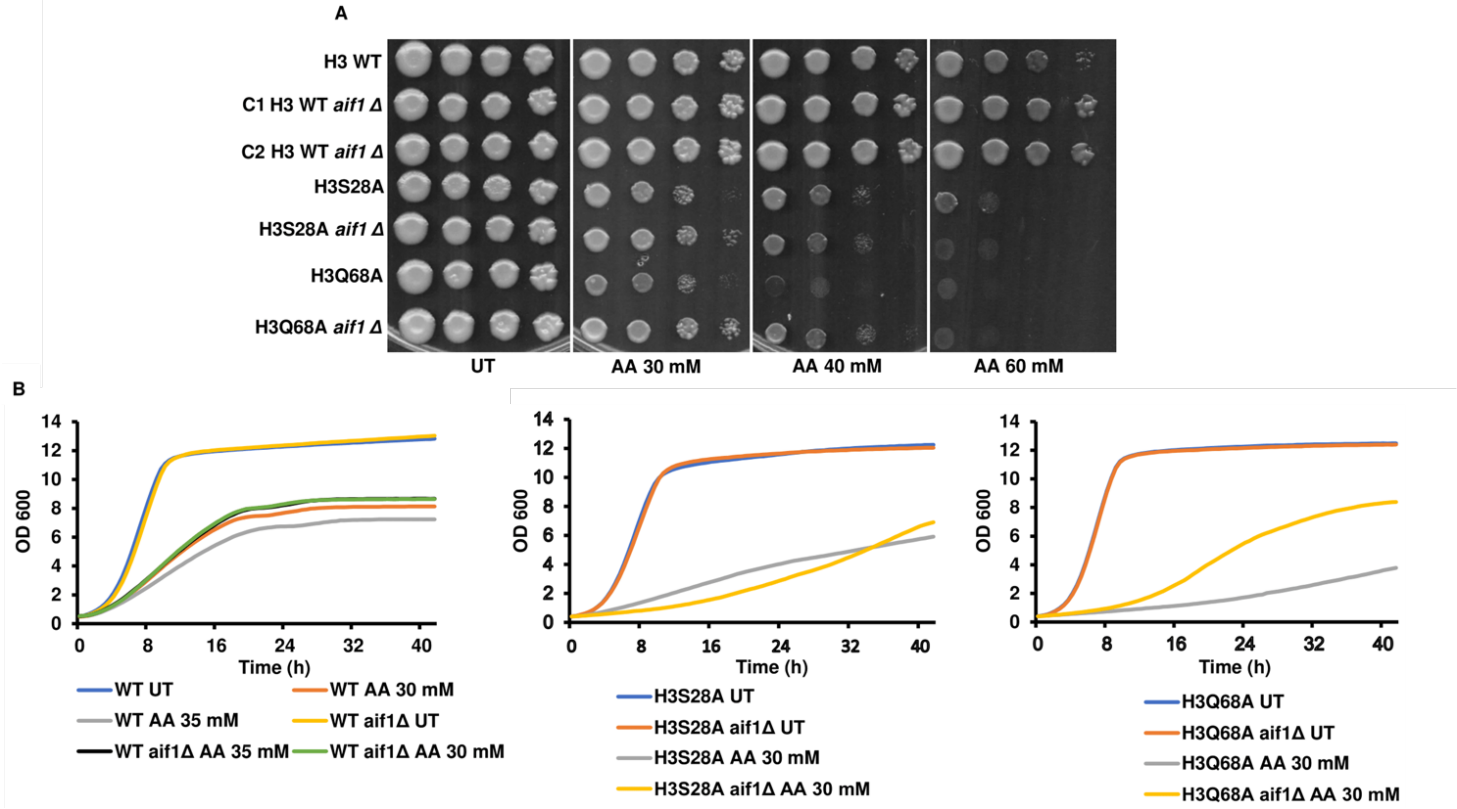
FIGURE 3: Deleting *aif1* in the acetic acid-sensitive mutants increases their survival to different degrees in acetic acid. **(A)** *aif1Δ* produced in the wild-type (H3 WT), H3S28A or H3Q68A were spotted in YPD pH 4.5 only (untreated) or different concentrations of acetic acid (30, 40, 60 mM). Cells were grown at 30 °C for 72h and scanned. **(B)** the cells used in panel A were grown in liquid culture under untreated (UT) or 30 mM acetic acid treated (AA) conditions. The graphs reflect the absorbance values at 600 nm at every 30' for the hours indicated in the x-axis.

Wissing *et al.*, 2004 have reported that *aif1Δ* cells exhibited significantly increased survival compared to their isogenic counterparts when exposed to apoptotic insult by hydrogen peroxide or acetic acid [17]. We reasoned that if enhanced transcript levels of *AIF1* are responsible for the sensitivity of the mutant strains towards acetic acid, then the gene deletion should increase resistance to the agent. Indeed, as reported, *aif1Δ* in the WT background increased the resistance to acetic acid. In the H3S28A *aif1Δ* strain, there was only a modest increase in the survival of the cells. On the contrary, the H3Q68A *aif1Δ* strain showed a pronounced increase in survival compared to H3Q68A (**Fig. 3A, B**). However, in the *aif1Δ* mutants, the growth defect was not wholly rescued, suggesting that other molecules within the mutant strains play important roles in acetic acid toxicity. We were unable to generate *aif1Δ* in other mutant backgrounds even after repeated attempts. Thus, we conclude that the *aif1Δ* partially rescues acetic acid sensitivity of the mutant strains.

### Acetic acid-sensitive mutants show chromatin fragmentation, distinct actin cytoskeleton distribution and enhanced ROS generation after treatment with acetic acid

**Figure 4:**
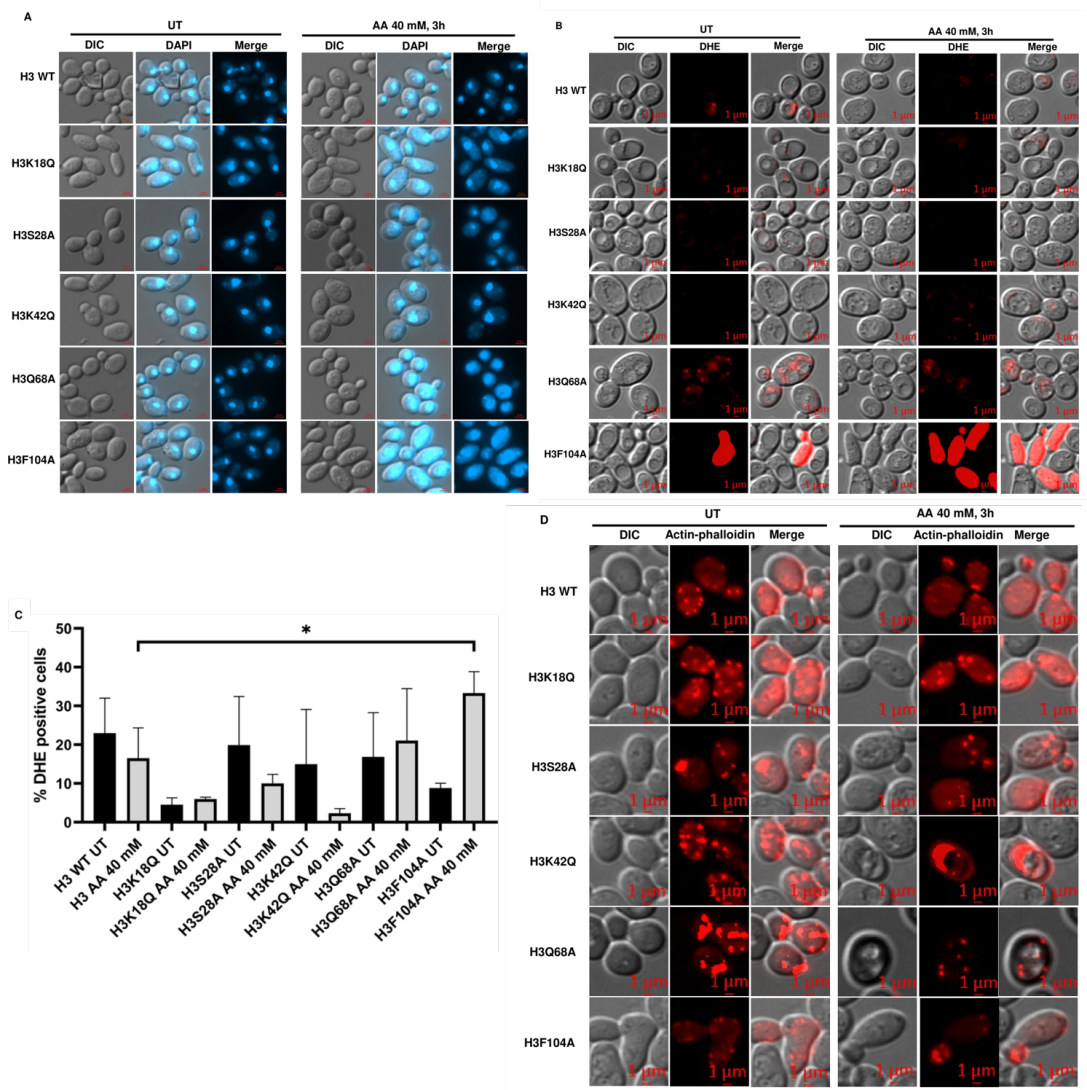
FIGURE 4: Acetic acid-sensitive mutants exhibit chromatin fragmentation, enhanced ROS generation and aggregated actin cytoskeleton after treatment with acetic acid. **(A)** DAPI **(B)** DHE (for ROS) **(C)** Quantification of DHE+ cells. **(D)** Actin-Phalloidin (for actin cytoskeleton) staining images of H3 WT and the mutant cells, which were grown till an absorbance of ∼1 at 600 nm and then left untreated (UT) or treated with acetic acid for the indicated time and concentration. ***p < 0.001, **p (0.001 to 0.01), *p (0.01 to 0.05).

Acetic acid treatment causes cell death through DNA damage [18] and ROS generation [4, 19]. 4',6-diamidino-2-phenylindole (DAPI) staining of a yeast mutant strain in the cell division cycle gene *CDC48* undergoing apoptosis has shown that chromatin fragments align in a ring-like structure near the nuclear envelope or are distributed throughout the cells [3]. Acetic acid treatment at a lower concentration of 20 mM for 3h revealed that the mutant H3Q68A shows the presence of randomly distributed chromatin fragments (Fig. S4A). Upon staining the histone mutants in the absence or presence of 40 mM acetic acid for 3h, we observed chromatin fragments in the mutants H3Q68A and H3F104A (**Fig. 4A**). Thus, the DAPI staining pattern reveals that the mutants might be undergoing apoptosis upon the acetic acid treatment. Cells exhibiting acetic acid induced RCD have enhanced levels of ROS as shown by staining with MitoTracker CM-H2-XRos [19]. Thus, we checked the status of ROS generation by dihydroethidium (DHE) staining in the WT and the mutants after 40 mM acetic acid treatment for 3h and found significantly higher ROS in H3F104A compared to the WT (**Fig. 4B, C**). Additionally, under prolonged treatment of 20 mM acetic acid for 6h, the H3K18Q and H3F104A strains showed significantly more ROS than the WT strain (Fig. S4B, C). Since hydrogen peroxide and menadione are oxidative stress-inducing agents, we probed into the sensitivity of the WT and mutant strains towards these ROS-generating agents [20]. As shown in Fig. S5A, B, H3F104A is sensitive to H_2_O_2_ and menadione. Thus, our DHE staining data corroborates with the high oxidative stress in the H3F104A mutant. Several cellular processes utilize the actin cytoskeleton dynamics. Recently, studies have established that treating jasplakinolide, an apoptotic agent stabilizes the F-actin in mammalian cells [21]. Jasplakinolide has also been shown to cause cell death in yeast cells [22]. Furthermore, aged apoptotic yeast cells exhibit an aggregated form of the actin cytoskeleton. Put together, actin dynamics is an important factor in deciding the commitment of the cells towards apoptosis. Thus, we stained the WT and mutant cells with actin phalloidin. **Fig. 4D** shows that at an acetic acid concentration of 40 mM for 3h, none of the mutant cells exhibit any change in actin phalloidin staining. However, at an acetic acid concentration of 20 mM for 6h, mutant cells H3S28A and H3K42Q show a distinctly different distribution of actin compared to the WT cells, which show a typical punctate pattern (Fig. S4D). Actin staining of the mutant acetic acid-treated cells shows a pattern reported for the aggregated actin cytoskeleton [22, 23]. Thus, we conclude that the oxidative stress, DNA damage and aberrant actin distribution may contribute to cell death of histone mutant(s) upon prolonged acetic acid treatment by exacerbating the acetic acid stress in these mutants [23].

### An enhanced basal level of *AIF1* correlates with the shortened CLS in the mutants

**Figure 5:**
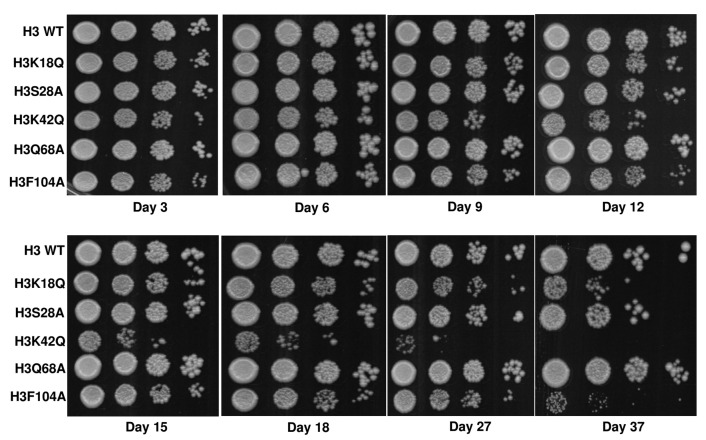
FIGURE 5: Chronological life span assay with wild-type (H3 WT) and mutants revealed that the mutants exhibit shortened life span. Spot-test assay of the wild-type (H3 WT) and mutant samples collected from the indicated days. The dried spots were incubated for 2 days and photographed.

The *aif1Δ* cell exhibits better survival than its WT in the CLS assay [17]. Prolonged yeast cultivation leads to cell ageing, which results in apoptosis [9]. The primary ageing mechanism in yeast cells is a consequence of acetic acid accumulation in the stationary phase culture [24]. We reasoned that if the mutants are sensitive to acetic acid and exhibited enhanced expression levels of the *AIF1* gene in the untreated condition, then these two factors may lead to cell death triggered through apoptosis during the prolonged cultivation of the cells. We grew an equal number of WT and mutant cells and checked for their survival on indicated days. As shown in **Fig. 5**, mutants H3K18Q, H3K42Q and H3F104A exhibited shortened CLS compared to the WT cells, which correlates with increased expression levels of the *AIF1* and sensitivity of these cells to acetic acid. The mutant H3Q68A does not exhibit significantly high gene expression levels, and the CLS data shows that it has lengthened survival. Together, our data suggest that increased expression levels of *AIF1* in the mutants might be a contributing factor to their shortened CLS as the prolonged cultivation of the cells also serves as an apoptotic insult and, therefore, triggers cell death in mutants with high expression levels of *AIF1* (**Fig. 5**).

### Acetic acid-sensitive mutant strains exhibit increased *RLM1, PRM5* expression levels, Slt2 phosphorylation and sensitivity to cell wall stress agents

**Figure 6:**
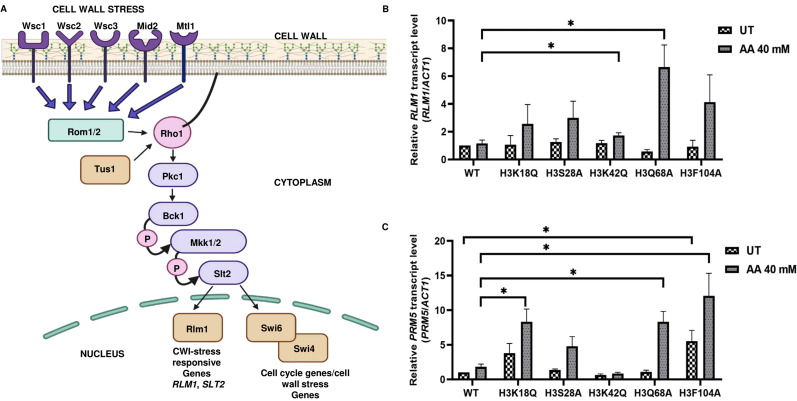
FIGURE 6: Acetic acid-sensitive mutants exhibit increased *RLM1* and *PRM5* expression levels indicating the involvement of the cell wall integrity pathway. **(A)** Schematic of cell wall stress response pathway in *S. cerevisiae*. **(B)** *RLM1* or **(C)** *PRM5* mRNA level was examined in the H3 wild-type (WT), and mutants under untreated (UT) or acetic acid treated (AA 40 mM) for 6h, the relative expression with respect to *ACT1* gene is shown. Statistical analysis was performed through unpaired Student's t-test with Welch's correction (GraphPad Prism 8 GraphPad Software, Inc). The mean ± SD of three biological replicates (n = 3) are shown. ***p < 0.001, **p (0.001 to 0.01), *p (0.01 to 0.05).

**Figure 7:**
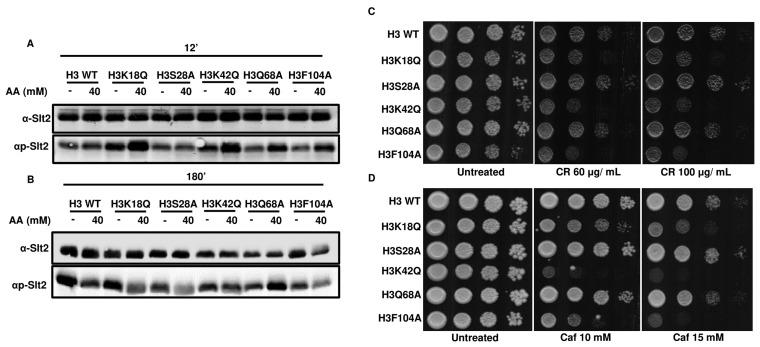
FIGURE 7: Activation of the Slt2 pathway is determined through phosphorylation in acetic acid-treated cells. **(A)** Protein extracts prepared from the indicated strains untreated or treated with acetic acid (40 mM) for 12'. Upper panel, immunoblots of wild-type **(**H3 WT) and mutant extracts with anti-Slt2 antibodies (control). Bottom panel, immunoblots of H3 WT and mutant extracts with antiphospho-p44/42 Slt2 antibodies. (**B)** Same as in **A** but with a treatment duration of 180'. **(C)** H3 WT and the mutants were spotted in YPD only (untreated) or different concentrations of Congo red (CR) (60, 100 μg/mL) or **(D)** Caffeine (Caf) (10, 15 mM). The spotted cells were grown at 30°C for 48h (CR) or 72h (Caf) and scanned.

Studies by Rego *et al.* showed that mating pheromone response and cell wall integrity pathways (**Fig. 6A**) are mediators of acetic acid-induced apoptosis [10]. In this study, an analysis of the downstream genes of the Rlm1p transcription factor in acetic acid revealed that the reduced levels of cell wall remodeling led to resistance against the apoptotic insult [10]. Furthermore, *BCK1* overexpression-mediated activation of the CWI pathway leads to enhanced sensitivity towards acetic acid. In contrast, strains generated by deleting genes belonging to the CWI pathway such as *bck1Δ* or *rlm1Δ* exhibited resistance against acetic acid [10]. In a separate study, Ribeiro *et al.* uncovered the yeast adaptive responses to acetic acid stress wherein the WT cells challenged with acetic acid showed a decrease in the expression levels of the chief transcription factor Rlm1 as well as the downstream targets, including *CHS3* and *PRM5* [11, 25, 26]. We analyzed the expression levels of *RLM1* and *PRM5* in acetic acid and untreated conditions and found that the mutants, unlike the WT, showed increased expression levels of *RLM1*. Compared to the WT cells, acetic acid treatment at 40 mM for 6 h increased expression levels to almost 6 times in the H3Q68A strain or 1.5 times in the H3K42Q strain (**Fig. 6B**). Similarly, *the PRM5* expression level, which marks the activation of the CWI pathway and is a target of *RLM1*, was significantly upregulated in the mutants H3K18Q (4.6 times), H3Q68A (4.6 times) and H3F104A (6.6 times) compared to the WT cells treated under similar conditions. Furthermore, H3F104A cells, even under untreated conditions, exhibited significantly higher expression levels of *PRM5* compared to the WT (**Fig. 6C**). We also checked the levels of other genes in the cell wall integrity pathway. However, they did not exhibit marked changes like the *PRM5* gene (Fig. S6). At pH 4.5, acetate strongly induces Slt2-phosphorylation in the yeast cells [27, 28]. We, therefore, tested the status of Slt2-phosphorylation post addition of acetic acid and found that at 12' after the addition of 40 mM acetic acid, all mutants except H3S28A and the WT exhibited increased Slt2-phosphorylation levels compared to their untreated condition (**Fig. 7A**). At 40 mM acetic acid treatment for 3h, we found that the mutant H3Q68A still showed phosphorylation indicating prolonged stress on its cell wall (**Fig. 7B**). Additionally, we repeatedly obtained more Slt2 phosphorylation of WT at the 3h time point but not at 12', indicating that the WT faces cell wall stress much later than the mutants, as most show increased phosphorylation at the initial time (12'). Cell wall damage in yeast cells leads to Slt2 protein activation, increasing its phosphorylation [29]. Since the increased Slt2-phosphorylation pointed to the pre-existing cell wall structure defect in the mutants, we analyzed if the mutants had any defect in the cell wall integration pathway by spotting them in the cell wall stress agents such as Congo red (CR) [30] or caffeine (Caf) [31] or 37°C [32]. We found that three mutants, H3K18Q, H3K42Q and H3F104A, are sensitive to both the stresses at 60 μg/mL and 100 μg/mL in CR and 10 mM and 15 mM in Caf (**Fig. 7C, D**) and only H3F104A is sensitive to growth at 37°C (Fig. S5B). Thus, our results strongly support enhanced acetic acid-mediated cell wall stress response in the mutants compared to the WT, possibly due to pre-existing cell wall stress.

### Acetic acid-sensitive mutants exhibit sensitivity towards disruption of ER, oxidative or metabolic homeostasis

**Figure 8:**
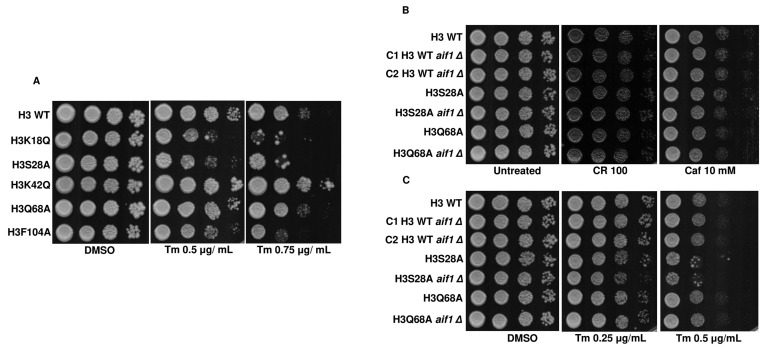
FIGURE 8: Acetic acid-sensitive mutants exhibit sensitivity towards disruption of ER homeostasis. **(A)** Spot-test assays of the wild-type (H3 WT) and mutants in the presence of different concentrations of tunicamycin (Tm) (0.5 μg/mL, 0.75 μg/mL). **(B)** Spot-test assays of the indicated strains in YPD only (untreated) or Congo red (CR) (100 μg/mL) or B, Caffeine (Caf) (10 mM). The spotted cells were grown at 30°C for 48h (CR) or 72h (Caf) and scanned. **(C)** Spot-test assays of the indicated strains in YPD supplemented with DMSO, which serves as control (DMSO) or different concentrations of tunicamycin (Tm) (0.25 μg/mL, 0.5 μg/mL). Note: C1 and C2 refer to clones 1 and 2, respectively.

Acetic acid treatment induces ER stress and UPR in the yeast cells through the misfolded protein accumulation in the ER. Additionally, it leads to the activation of Hac1p, a transcription factor responsible for mediating ER stress and Ire1p, a sensor for ER stress [33]. Thus, the induction of the cell wall integrity genes upon treatment of the mutants with acetic acid and the increased Slt2-phosphorylation levels, along with the sensitivity of mutants to cell wall stress, implies that these mutants may be more prone to the acetic acid stress due to the induction of both the ER and cell wall stress by the molecule. To test this hypothesis, we spotted the cells in the presence of tunicamycin (Tm), an inhibitor of N-linked glycosylation and a well-known ER stress-causing agent **Fig. 8A**. The assay revealed that three mutants, H3K18Q, H3S28A and H3F104A, are sensitive to the agent, implying that these cells may be facing both the ER and cell wall stress because of acetic acid treatment. Additional experiments, such as the deletion of the *IRE1* [34] in the background of these mutants, would shed light if the ER stress pathway were majorly responsible for the sensitivity of these mutants to the acetic acid stress. We also probed into the involvement of Aif1p in ER stress or cell wall stress pathways by spotting the *aif1Δ* strains in the presence of Tm or CR or Caf. **Fig. 8B, C** shows that the deleted strains did not show differential survival in the presence of either agent, thus ruling out the possibility of Aif1 mediated effect on either of the pathways. Acetic acid-mediated stress response in yeast also includes the induction of *the ESA1* gene [35]. The gene product is involved in the autophagy of the yeast cells [36]. We checked the sensitivity of the histone mutants in the presence of autophagy inhibiting or inducing chloroquine or rapamycin drug [37, 38]. Fig. S7A, B and C show that the H3K18Q, H3K42Q and H3F104A strains showed sensitivity to both agents indicating that these mutants may have imbalanced autophagic responses, which may be involved in the sensitivity towards acetic acid stress. Finally, we checked the sensitivity of mutants in the presence of non-fermentable carbon sources such as ethanol (2%) and glycerol (2%) to check their mitochondrial function status [24, 39]. As shown in Fig. S5B, mutants H3K18Q, H3K42Q, and H3F104A showed sensitivity in ethanol-supplemented media, indicating that the mutants would have compromised mitochondrial respiration. However, H3K18Q and H3K42Q could grow in glycerol-supplemented media, which means that mitochondrial respiration is intact in these mutants. One of the reasons the mutants are sensitive to ethanol but not glycerol is probably due to the inability of these mutants to utilize 2-carbon source, ethanol, whereas they can utilize 3-carbon source, such as glycerol.

### Screening of N-terminal truncation mutants of histone H3 acetic acid identifies new hypersensitive mutants

**Figure 9:**
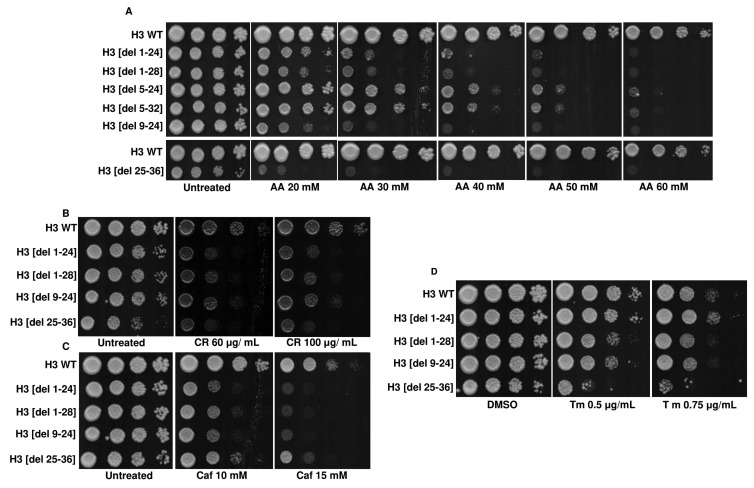
FIGURE 9: Screening of H3 N-terminal deletion mutants identifies acetic acid-hypersensitive mutants. **(A)** Spot-test assays of the wild-type (H3 WT) and N-terminal tail truncation mutants in different acetic acid concentrations (20, 30, 40, 50, 60 mM). Plates were incubated at 30°C for 72h and photographed. **(B)** Spot-test assays of the H3 WT and N-terminal tail truncation mutants in different concentrations of Congo red (CR) (60, 100 μg/mL) or **(C)** Caffeine (Caf) (10, 15 mM). Plates were incubated at 30 °C for 48h (CR) or 72h (Caf) and scanned. **(D)** Spot-test assays of the H3 WT and N-terminal tail truncation mutants in different concentrations of tunicamycin (Tm) (0.5 μg/mL, 0.75 μg/mL). Images were scanned after 72h.

Histone tails have multiple modifiable residues which are post-translationally modified. Such modifications change the fate of the associated nucleosomes in multiple ways [40]. For example, the N-tail acetylation of histone H3 regulates the chromatin assembly, which depends on DNA replication. Similarly, H3K4 methylation correlates with transcriptional activation. On the contrary, methylation on the H3K9 or H3K27 correlates with transcriptional silencing [40]. Some studies have also shown the involvement of H3 N-terminal tail residues in coping with the hydroxyurea insult. At the level of organization, the histone H3 N-terminal tail takes part in the sliding of the nucleosome, DNA wrapping within the nucleosome and the exchange of histone dimer [41]. Despite a plethora of information on the role of the H3 N-terminal tail residues, the importance of the residues in acetic acid tolerance has yet to be reported. Liu *et al.* screened the histone H3 point mutants in acetic acid. However, the study excluded histone H3 N-terminal tail truncations. To understand the role of histone H3 N-terminal tail residues in acetic acid, we screened the tail truncations in different concentrations of acetic acid. We found that at 40 mM or higher concentrations of acetic acid, almost all the N-terminal tail truncations are sensitive, underscoring the importance of N-terminal tail residues in the stress (Fig. S8A, B). Additionally, we found that four deletions viz., H3 [del 1-24], H3 [del 1-28], H3 [del 9-24] and H3 [del 25-36] exhibit the highest level of sensitivity to acetic acid (**Fig. 9A**). Furthermore, we tested if these deletions are sensitive to cell wall perturbing agents CR, Caf or 37°C and found that all of them are sensitive to CR and Caf at tested concentrations (**Fig. 9B, C**) but not in 37°C (Fig. S9B). Thus, we conclude that, like the point mutants, these tail truncations may exhibit enhanced cell wall integrity response in the presence of acetic acid.

### N-terminal tail truncated hypersensitive mutants exhibit sensitivity towards disruption of ER, oxidative or metabolic homeostasis

Through the screening of the H3 N-terminal tail truncation mutants in acetic acid, we identified a set of hypersensitive mutants. These acetic acid-sensitive tail truncation mutants failed to grow in the cell wall stress causing agents. So, we tested if these deletions were sensitive to the ER stress agent, Tm. On performing the spot test assay, we found that only one of the deletions, H3[del 25-36], failed to grow in the presence of Tm (**Fig. 9D**), which may contribute to acetic acid stress. Additionally, among the N-terminal tail truncation mutants, H3 [del 1-28], H3 [del 9-24], and H3 [del 25-36] are sensitive to H_2_O_2_. In contrast, in menadione, all the mutants were sensitive, indicating a poor system of tackling oxidative stress in the mutants, which may arise due to acetic acid stress (Fig. S9A, B). H3 [del 1-24] and H3 [del 9-24] showed compromised growth in ethanol-supplemented medium but not in glycerol, possibly because they can utilize 3-carbon but not 2-carbon sources. Alternatively, ethanol may present some less favourable stress for these sensitive cells (Fig. S9B).

## DISCUSSION

**Figure 10:**
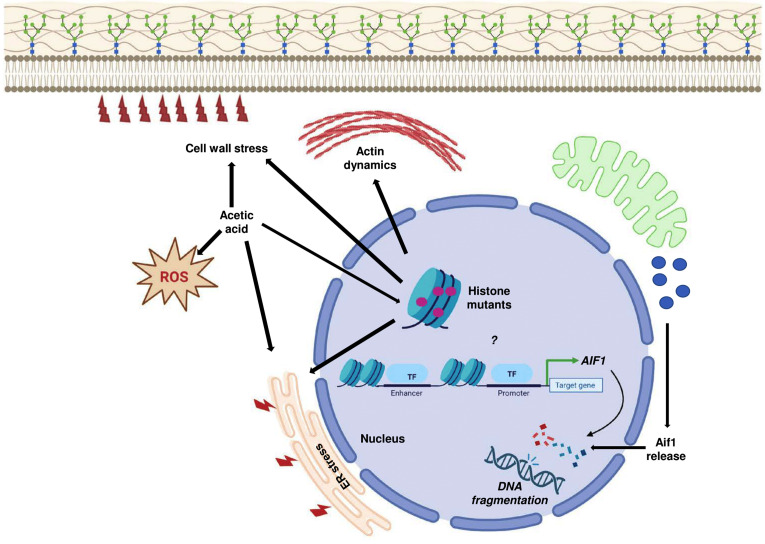
FIGURE 10: Acetic acid response in histone mutants. The sensitive histone mutants exhibit induced expression levels of *AIF1*, which probably reflects the increased apoptotic stress in the mutants after acetic acid treatment. Thus, the increased gene expression may be an indirect consequence of increased stress in the mutant cells or maybe because of the importance of the mutated histone residue in regulating the gene. Acetic acid-mediated ER and cell wall stress may exacerbate the effect on histone mutants with pre-existing sensitivity towards ER stress or cell wall stress. Pronounced stress by acetic acid in the mutants increases the ROS and pre-existing defective actin dynamics together with all the factors that cause sensitivity of the histone mutants in acetic acid.

Yeast cells respond to acetic acid in diverse ways [4]. Here, we have elucidated the deleterious effects of acetic acid on different histone mutants. As has been shown through our results, though some of the histone mutants are sensitive to acetic acid, they exhibit different ways of responding to the same concentration of the molecule, which underscores the genetic diversity of these strains (**Fig. 10**). We show that the mutants have enhanced basal expression levels of critical proapoptotic molecule *AIF1* which is involved in the DNA fragmentation in the process of apoptosis. One of the mutants, H3Q68A, did not show a pronounced gene expression level. However, deletion of the gene in the mutant resulted in significant rescue in acetic acid. These results appear contradictory. Thus, we suggest, although the expression levels of the gene remain unaltered in the mutant, the Aif1 protein may be majorly responsible for its sensitivity, and the translocation of the protein from the mitochondria to the nucleus may be extremely fast in the presence of acetic acid. On the contrary, in H3S28A cells with significantly high expression levels of the *AIF1* gene in acetic acid stress, the deletion of *aif1* did not increase survival appreciatively, which may be because the sensitivity of these cells is not majorly dependent on the protein function but on some other molecule.

Two mutants in the acetic acid-sensitive strains list are H3K18Q and H3K42Q. Both the residues, H3K18 and H3K42, are important sites for acetylation, known to mediate various cellular processes. Thus, the regulation of acetylation on these residues may serve as an important determinant of acetic acid sensitivity.

H3K18 residue acetylation is associated with mid-log transcriptional rate and is involved in many transcriptional changes. It is usually associated with histone modifications such as H3K4me3. In the case of diamide stress, the acetylation mark is rapidly lost at the +1-nucleosome position of the repressed ribosomal biogenesis genes. On the contrary, the induced genes under stress have nucleosomes enriched in acetylation marks of H3K18. Thus, the modification plays a vital role in eukaryotic transcriptional regulation [42]. Since the H3K18Q mimics the acetylated state of the lysine residue, it may lead to nucleosome displacement at the *AIF1* gene promoter to induce its expression at the basal level. In a different scenario, H3K18Ac shows links to the replicative life span of yeast, wherein cells with inhibited *GCN5* exhibited an increased life span via its effect on the acetylation of H3K9 and 18 residues [43]. Interestingly, the transcriptome analysis showed the involvement of *ADA2* in acetic acid stress response. The study showed that overexpression or deletion of *ada2* makes cells more sensitive to acetic acid stress [35]. Also, *ada2* deletion abolishes Gcn5 activity primarily involved in H3K18Ac [44]. Modifiable lysine in H3K18 residue also plays a vital role in maintaining the longevity of the cells since the mutant form H3K18Q exhibits shortened CLS, which corroborates our result.

Studies have shown that in the presence of hydrogen peroxide or acetic acid, the acetylation of H2BK11 is removed by Hos3 histone deacetylase (HDAC). The acetylated variant H2BK11Q is resistant to the apoptotic insult by H_2_O_2_, whereas the deacetylated mimic H2BK11R is sensitive [45]. In another study, deletions of Set3 HDAC complex components viz., *HOS2, SNT1, CPR1, HST1, SIF2, SET3, HOS4* as well as *HOS3* show resistance to acetic acid, indicating critical involvement of histone acetylation marks in resistance against acetic acid [46].

H3S28A mutant shows increased nucleosome occupancy at the telomeric region. Telomeres have a repressive chromatin environment associated with late replication initiation at the origin [47]. Thus, the histone residue may be crucial in maintaining replication initiation at the origin. In mammalian systems, the H3S28ph mark is associated with stress-responsive gene activation in response to the activation of the MAP kinase pathway [48].

Within the nucleosome structure, H3K42 serves as a transcriptional “gatekeeper”, which plays a role in transcriptional attenuation. At the DNA entry/exit position, this residue serves as the sole point of contact of the nucleosome. H3K42 undergoes dimethylation to convert into H3K42me2. The mutation in H3K42, such as H3K42A, disrupts the methylation of the residue and is sensitive to multiple DNA damaging agents such as methyl methanesulphonate (MMS) and hydroxyurea (HU), microtubule-destabilizing molecule, benomyl, and a transcriptional inhibitor, 6-azauracil (6AU) [49]. Another mutant, H3K42Q, is also a non-methylated mimic of H3K42 and is sensitive to 6-AU. Transcriptional profiling of both the mutants, viz., H3K42A or H3K42Q, revealed that they alter the genome expression by majorly up-regulating the genes. Thus, H3K42A is less restrictive towards the elongating RNAPII complex leading to increased efficiency of the elongation and the similar transcriptional profile of the non-methylated mimic H3K42Q points to a similar mechanism for observed upregulation of many genes [49]. Our results show significant up-regulation of *the AIF1* gene in the H3K42Q mutant under untreated and treated conditions. Thus, the uninhibited transcription results in the upregulation of the gene(s), making the mutant sensitive to acetic acid.

An Spt assay with histone mutants to reveal the residues involved in DNA-mediated reactions showed that H3Q68 has an Spt-phenotype [50]. Spt assay reveals the effect of a mutation on transcription. It is based on the insertion of the yeast Ty element, which disrupts gene transcription. Any mutant which results in a negative phenotype can restore the gene transcription. Thus, the H3Q68 mutation may affect the transcription of genes. H3Q68 is positioned in the region of DNA interaction and may play a role in cell growth or DNA repair [51]. Since acetic acid causes DNA damage, the residue may play an indirect role in curbing its adverse effects by repairing the damage. In another hypothesis, the DNA region associated with the residue may be critically involved in tolerance to acetic acid.

H3F104 residue is present in the globular region. H3F104A mutant was uncovered in a screen to identify histone mutants which reduce the gene expression levels of histone H3 and H4 [52]. Reduced histone protein levels of H3 or H4 are implicated in shorter CLS [52]. Both histone H3 and H4 protein levels were less in the H3F104A mutant. Thus, in line with the hypothesis, H3F104A exhibits shortened CLS [52]. The H3F104 residue also plays a role in the translocation of DNA by the SWI/SNF complex [53] and quiescence [54] [55]. Altogether, the H3F104 residue is crucial for adequately maintaining cellular functions. Our data corroborate the previous results as H3F104A exhibits shortened CLS and reduced viability in all the stress conditions we have used (due to reduced protein levels of H3 and H4 which can affect a plethora of cellular functions), thereby underscoring the importance of the residue in a variety of pathways involved in cellular homeostasis maintenance.

The N-terminal tail of histone H3 protrudes out of the nucleosome and is, therefore, more available for histone modifications. The residues at the N-terminal tail undergo various modifications such as acetylation, methylation etc., which play crucial roles in cellular processes. For example, acetylation at 9 and 14 of H3 lysine residues occur post-UV irradiation. The H3 acetylation aids in the SWI/SNF remodeler recruitment, removing UV-induced lesions [56]. Another example includes lysine methylation at the fourth position of histone H3, which plays a role in the S-phase checkpoint of the yeast. Furthermore, H3 N-terminal tail residues are crucial for the DNA damage response, base excision repair (for single-stranded DNA breaks), the phosphorylation of Rad53, and maintenance of nucleosome stability [41, 56]. Acetic acid treatment results in single-stranded DNA breaks (SSBs) [18]. Over time, if unrepaired, the SSB can give rise to double-strand DNA breaks (DSBs). These lesions are repaired using a non-homologous end-joining (NHEJ) pathway [56]. Many of our hypersensitive histone H3 N-terminal tail truncations are defective in NHEJ viz., H3 [del1-24], H3 [del1-28] and H3 [del9-24] [13]. Thus, it may happen that since these mutants already have compromised single-stranded DNA break repair pathway, the accumulation of SSBs post acetic acid treatment may lead to DNA double breaks, and due to the defective NHEJ in the tail-truncation mutants, they fail to repair the DNA leading to their death/cell arrest.

The revelation of acetic acid-induced cell death mechanisms in yeast can aid in the design of appropriate strategies to improve the fermentation by carefully targeting the genetic make-up in a manner such that the gene products involved in increasing the sensitivity is reduced, which ultimately would increase the efficiency of these strains in the fermentation process. Additionally, the cell death mechanisms elucidation by acetic acid can also provide impetus to developing strategies that decrease food spoilage by yeast species resistant to acetic acid.

## MATERIALS AND METHODS

### Culture conditions

Yeast strains (Table S1) were cultured at 30°C and 200 rpm shaking condition maintained in the Brunswick incubator shaker. The strains were regularly maintained in normal YPD (1% yeast extract, 2% mycological peptone, 2% dextrose, 2% agar) or SC-Leu. For studying the response of acetic acid stress on mutants, the library created by the Boeke group was utilized [13]. Since acetic acid remains in half-dissociated and half-undissociated form at low pH of 4.5, which ensures better diffusion of the weak acid into the cells, the experiments were performed in YPD with pH maintained at 4.5 (using HCl) before autoclaving [14]. Glacial acetic acid was used to prepare a stock of concentrated acetic acid (8.7 M, pH 4.5). Different concentrations of acetic acid used in the study were directly obtained by adding acetic acid from the stock solution.

### Growth assays

Yeast strains were tested in various conditions using either drop-test assays or growth curves, as detailed below:

#### Drop-test assays

The saturated yeast cultures grown overnight were harvested, and an equal number of cells (with an absorbance of 1 at 600 nm) were serially diluted 10-fold from the initial culture. Equal numbers of cells were spotted on plates. The spots were air-dried and incubated at an optimal temperature of 30°C.

#### Growth Curve Analysis

Growth curve analysis was used to reconfirm the phenotypes obtained in the spot test assay. Overnight grown cells were diluted to an absorbance of 0.2 at 600 nm and allowed to grow till the exponential phase. Cells at the exponential phase were diluted to 0.2 OD using the fresh medium, left untreated or treated with an appropriate agent, and seeded into 96 well plates (Nunc). These plates were incubated in the plate reader set to measure OD at 600 nm at an interval of 30 min.

### Chronological life span (CLS) assay

CLS was performed as described previously [9] with certain modifications. Saturated cultures of H3 WT and mutants were serially diluted, and aliquots containing an equal number of cells (1000) were plated on YPD agar and incubated for 2-3 days at 30°C. Colonies were counted at the end of the incubation period, and cellular viability percent was determined for the indicated number of days. Parallelly, drop-test assays were performed with the same cultures.

### Protein extraction (Trichloroacetic acid (TCA) precipitation method)

Whole-cell extracts were prepared from yeast cells using the TCA method [57] with few alterations. Briefly, the yeast cells harvested under untreated/treated conditions were washed using 20% TCA solution and kept at -80°C until further use. Cell pellets were resuspended in 20% TCA and glass beads for extraction, which were then lysed through vigorous vortexing. The precipitated protein was centrifuged at 7000 rpm. The supernatant was decanted, and the pellet washing with Tris-Cl (pH 7.5) was performed. Finally, the pellet was boiled in 1× loading buffer. The supernatant containing the protein was used for the western blot.

### Western blotting

SDS-PAGE was employed to resolve the proteins which were transferred to the nitrocellulose membrane using the Tris–Cl (pH 7.5), glycine, methanol and SDS-based transfer buffer. After the transfer, the membrane was blocked with 2.5% bovine serum albumin (BSA) (catalogue no.: MB083; HiMedia). The membrane was incubated in the primary antibody for 90 min and then washed. Next, the membrane was incubated in a secondary antibody for 45 min and washed. Finally, the dried membrane was visualized using LI-COR infrared imaging system. Primary and secondary antibodies used for immunoblotting were as follows: α-TBP1 polyclonal antiserum from rabbit (generated in the laboratory), anti-phospho-p44/42 (Cell Signaling, catalogue no. 4370S), anti-Mpk1 (Santa Cruz Biotechnology, Inc., catalogue no. SC-6803), goat anti-rabbit IgG secondary antibody (catalogue no.: A32734; Invitrogen) and rabbit anti-goat (catalogue no.: A21088; Invitrogen).

### DNA isolation from yeast

After overnight culture, cells were harvested and subjected to DNA extraction using the established method [58]. Briefly, the cell pellet was mixed with 0.2% SDS (300 μL), vortexed, and boiled for 15 min at 100°C. NaCl was added to a final concentration of 100 mM. The contents were briefly vortexed and centrifuged at 10,000 rpm for 15 min. The supernatant was transferred to a new vial. The resulting lysate was mixed with an equal volume of phenol (pH 8): chloroform: isoamyl alcohol (25:24:1) and centrifuged for 15 min at 13,000 rpm. DNA containing aqueous phase was added to a fresh tube with absolute ethanol and incubated for 30 min at -80°C. DNA obtained post centrifugation was air dried and resuspended in MQ and transferred to 4°C. For the semiquantitative PCR, the manufacturer's instructions (KAPA Taq DNA Polymerase; catalogue no.: KK1015) were followed to check the amplification of different genes.

### RNA extraction and quantitative real-time PCR of genes

Primary cultures of strains were secondary cultured at 0.2 absorbance and grown till the exponential phase. The untreated/treated cultures were harvested, and RNA extraction was done using the hot phenol method [59]. RNA samples were normalized to the same concentration of 250 ng/μL to synthesize the complementary DNA using iScript cDNA synthesis kit (catalogue no.: 1708891; Bio-Rad). RT–PCRs of respective genes were performed using ABI-7300 RT–PCR with Sequence Detection System v1.4 (Applied Biosystems). Relative mRNA expression was calculated using the 2-ΔΔCT method [60]. The relative expression fold changes were calculated using control as the *ACT1* gene. Data are represented as mean ± SD of three biological replicates. Complete list of primers is summarized in Table S2.

### Fluorescence microscopy

#### 1. Actin-Phalloidin or AP staining

Actin-Phalloidin staining was performed as described previously [61]. Briefly, the cells were harvested at 4,000 rpm for 90 s and washed with 1XPBS. These cells were fixed with 4% formaldehyde for 1 h at RT. The fixed cells were washed twice with 1XPBS, and the cells were resuspended in 1XPBS containing (100 μM) actin-phalloidin stain (catalogue no.: P1951) and incubated at 30°C for 1 h in the dark. The cells were harvested and visualized under the fluorescence microscope.

#### 2. Dihydroethidium (DHE) staining

DHE staining to assess ROS was performed using an established protocol [62]. Briefly, Cells untreated or treated were harvested by centrifugation at 4,000 rpm for 3 min. Cells were then washed once with 1XPBS. The washed cells were resuspended in PBS containing 50 μM DHE (Sigma Aldrich, catalogue no.: D7008) and incubated for 1 h at 30°C. Post incubation, the cells were centrifuged at 4,000 rpm for 3 min at 4°C, washed twice with 1XPBS and visualized using the fluorescence microscope with a 63× objective lens.

#### 3. 4',6-diamidino-2-phenylindole (DAPI) staining

DAPI staining was performed using a previously established protocol [62]. Briefly, cells were collected by centrifugation at 3,000 rpm for 2 min at 4°C. The pellet was washed once with 1XPBS, resuspended in 70% ethanol, and incubated at -80°C for 1 h. Post incubation, the cells were centrifuged at 3,000 rpm for 2 min and washed once with 1XPBS. The pellet was resuspended in PBS containing DAPI (1 μg/mL), stained for an hour at 4°C, and visualized under the fluorescence microscope.

### Transformation and gene knockout

#### 1. Competent cell preparation

Yeast cells were inoculated in YPD media from the plate and grown overnight at 30°C. The next day the cells were diluted to a 0.2 OD in 50 mL YPD and grown to 0.8 – 1 OD, after which cells were harvested and washed with MQ and lithium acetate buffer. Finally, the cell pellet was resuspended in lithium acetate buffer and incubated at room temperature for 30 min.

#### 2. Gene knockout

The template for knocking out the *AIF1* gene was generated through 2-step PCR of pRS405 plasmid and the primers specific for *AIF1* KO [63]. The purified product was used to transform yeast cells [64], and the positive colonies were selected using SC-Leu plates. The knockout was confirmed through PCR amplification of the gene.

### Colony Forming Unit (CFU) Assay

CFU assay was performed to quantify yeast cells' survival in acetic acid's presence [65]. Briefly, cells were grown for the desired time in the absence or presence of acetic acid. Following the incubation, the cells were harvested and washed twice with distilled water. Finally, the cells were serially diluted and plated in equal numbers (1000). Values from three biological replicates were quantified.

## AUTHOR CONTRIBUTION

N.S., S.S., and R.S.T. conceptualization; N.S. and S.S. investigation, methodology, validation, and formal analysis; R.S.T supervision; N.S. writing-original draft; N.S. and R.S.T. writing – review & editing; R.S.T. project administration; N.S. and R.S.T. funding acquisition.

## SUPPLEMENTAL MATERIAL

Click here for supplemental data file.

All supplemental data for this article are available online at www.microbialcell.com/researcharticles/2023a-saha-microbial-cell/.

## Abbreviations:

PS: – phosphatidylserine,
RCD: – regulated cell death,
AA: – acetic acid,
WT: – wild-type,
CWI: – cell wall integrity,
Pkc1: – protein kinase C,
MAPK: – mitogen-activated protein kinase,
AIF1: – apoptosis inducing factor,
CLS: – chronological life span,
ROS: – reactive oxygen species,
H3: – histone 3,
Caf: – caffeine,
CR: – congo red,
slt2: – suppressor of the lytic phenotype,
DAPI: – 4',6-diamidino-2-phenylindole,
DHE: – dihydroethidium,
Tm: – tunicamycin,
HDAC: – Hos3 histone deacetylase,
MMS: – methanesulphonate,
HU: – hydroxyurea,
6AU: – 6-azauracil,
SSBs: – single-stranded DNA breaks.
