# Supplementary material for: Investigation of the acetic acid stress response in Saccharomyces cerevisiae with mutated H3 residues
Source: Microb Cell. 2023 Aug 18;10(10):217–32. doi: 10.15698/mic2023.10.806 (PMC10513452; doi:10.15698/mic2023.10.806)
Supplement: Supplementary file 1 [file mic-10-217-s01.pdf]

SUPPORTING INFORMATION

Investigation of the acetic acid stress response in *Saccharomyces cerevisiae* with mutated H3 residues

Nitu Saha[1], Swati Swagatika[1] and Raghuvir Singh Tomar[1,*]

[1] Laboratory of Chromatin Biology, Department of Biological Sciences, Indian Institute of Science Education and Research Bhopal, 462066, Madhya Pradesh, India.

* Corresponding Author:

Raghuvir Singh Tomar, Department of Biological Sciences, 219, Academic Building-3, Indian Institute of Science Education and Research Bhopal, Bhopal By-pass Road, Bhauri, Bhopal 462 066, Madhya Pradesh, India; Phone: +91 755 269 1411; Phone Lab: +91 755 269 1412; E-mail: rst@iiserb.ac.in

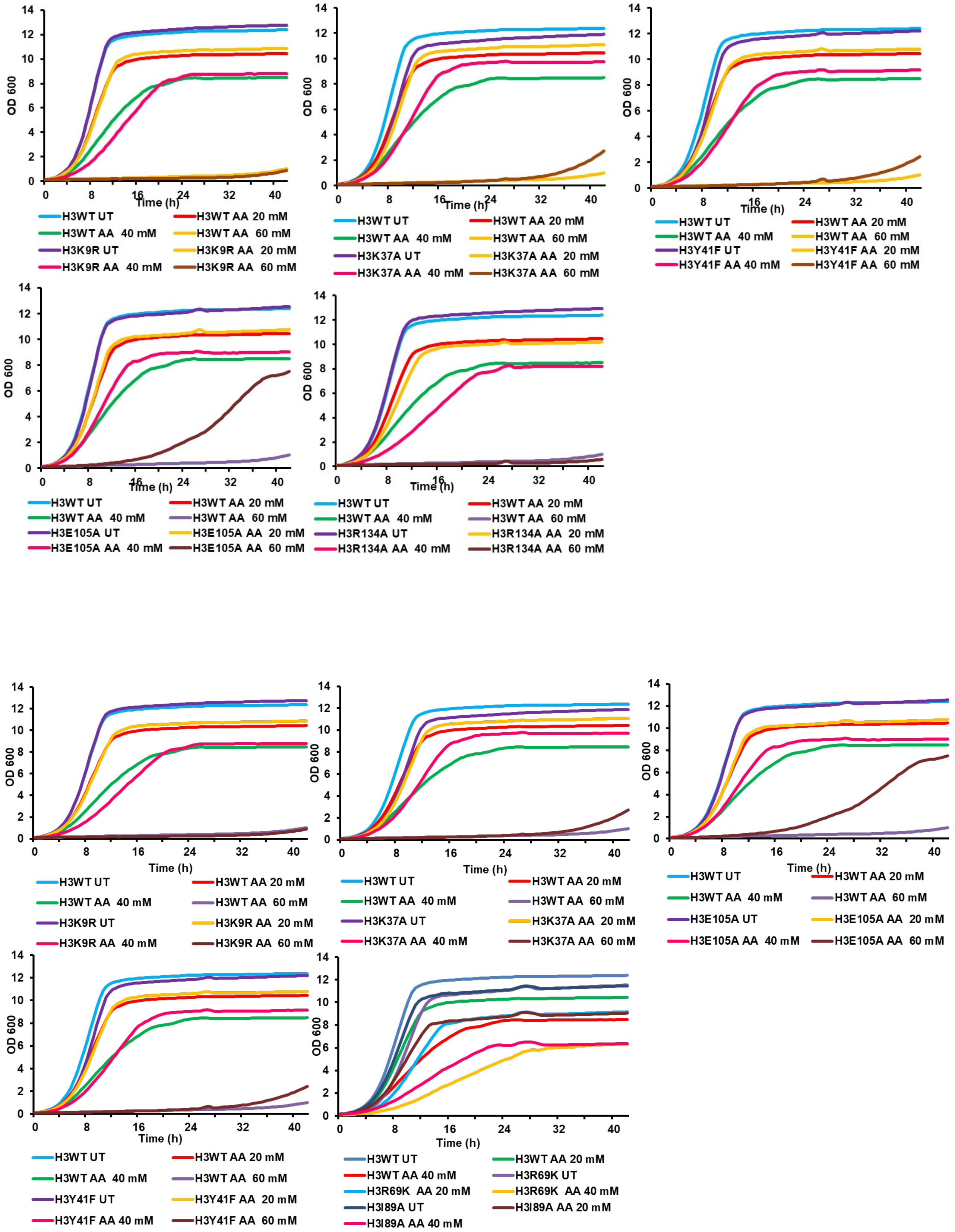

FIGURE S1: Growth curve assays for revalidation of the previously identified acetic acid-sensitive mutants. Growth of the wild-type (H3 WT) and indicated mutant cells in liquid culture under untreated (UT) or acetic acid treated (AA) (20, 40 and 60 mM) conditions. The graphs reflect the absorbance values at 600 nm at every 30’ for the hours indicated in the x-axis.

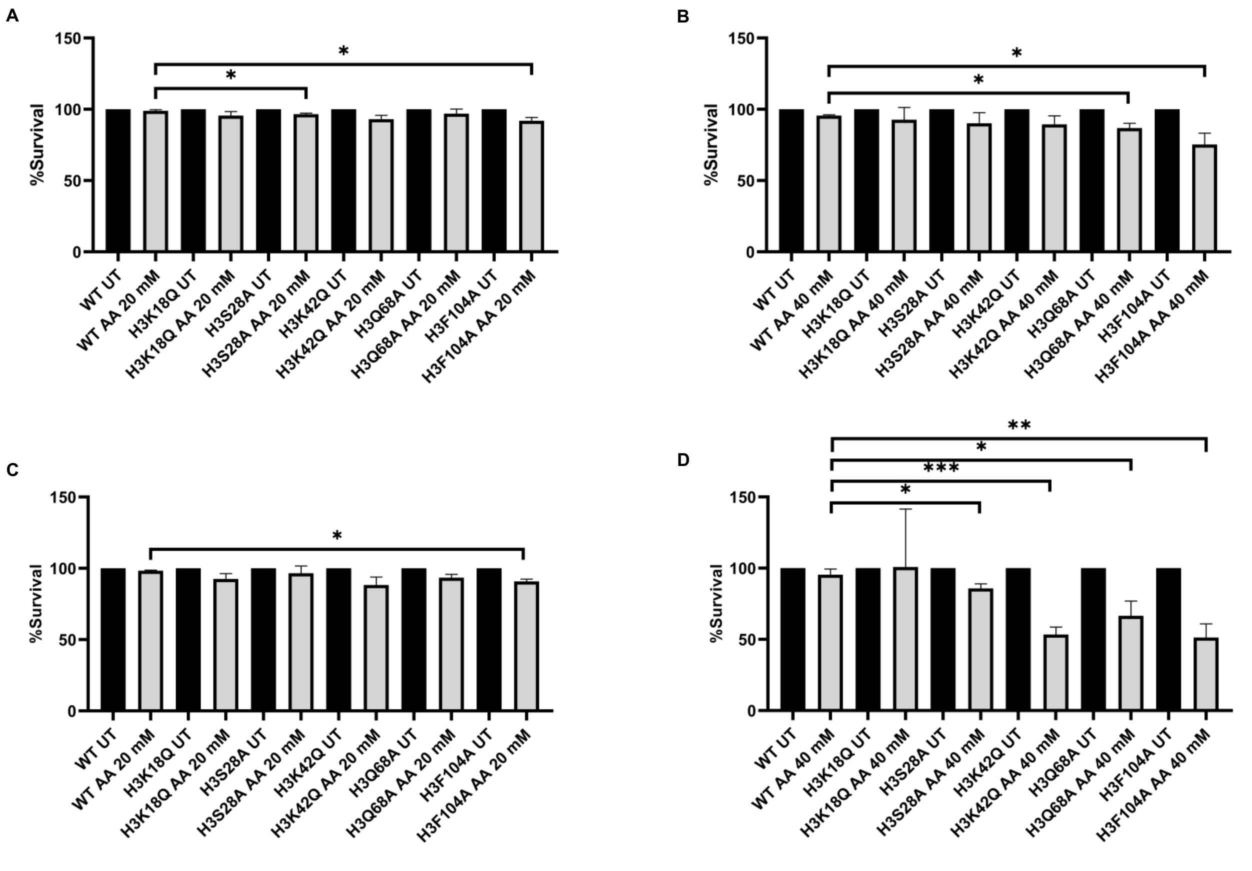

Figure S2. Survival of wild-type (H3 WT) and mutants in the presence of acetic acid. A, Survival graphs for the indicated strains grown in the absence or presence of 20 mM acetic acid for 90'. **B,** Same as **A** but cells were treated with 40 mM acetic acid. **C,** Same as **A** but cells were treated for 3h. **D,** Same as **C** but cells were treated with 40 mM acetic acid. Survival % of WT and mutants were calculated as [(number of cells in untreated or treated condition/ number of cells in untreated condition) *100] %. Statistical analysis was performed through unpaired Student's t-test with Welch's correction (GraphPad Prism 8 GraphPad Software, Inc). The mean ± SD of three biological replicates (n = 3) are shown. ∗∗∗p < 0.001, ∗∗p (0.001 to 0.01), ∗p (0.01 to 0.05).

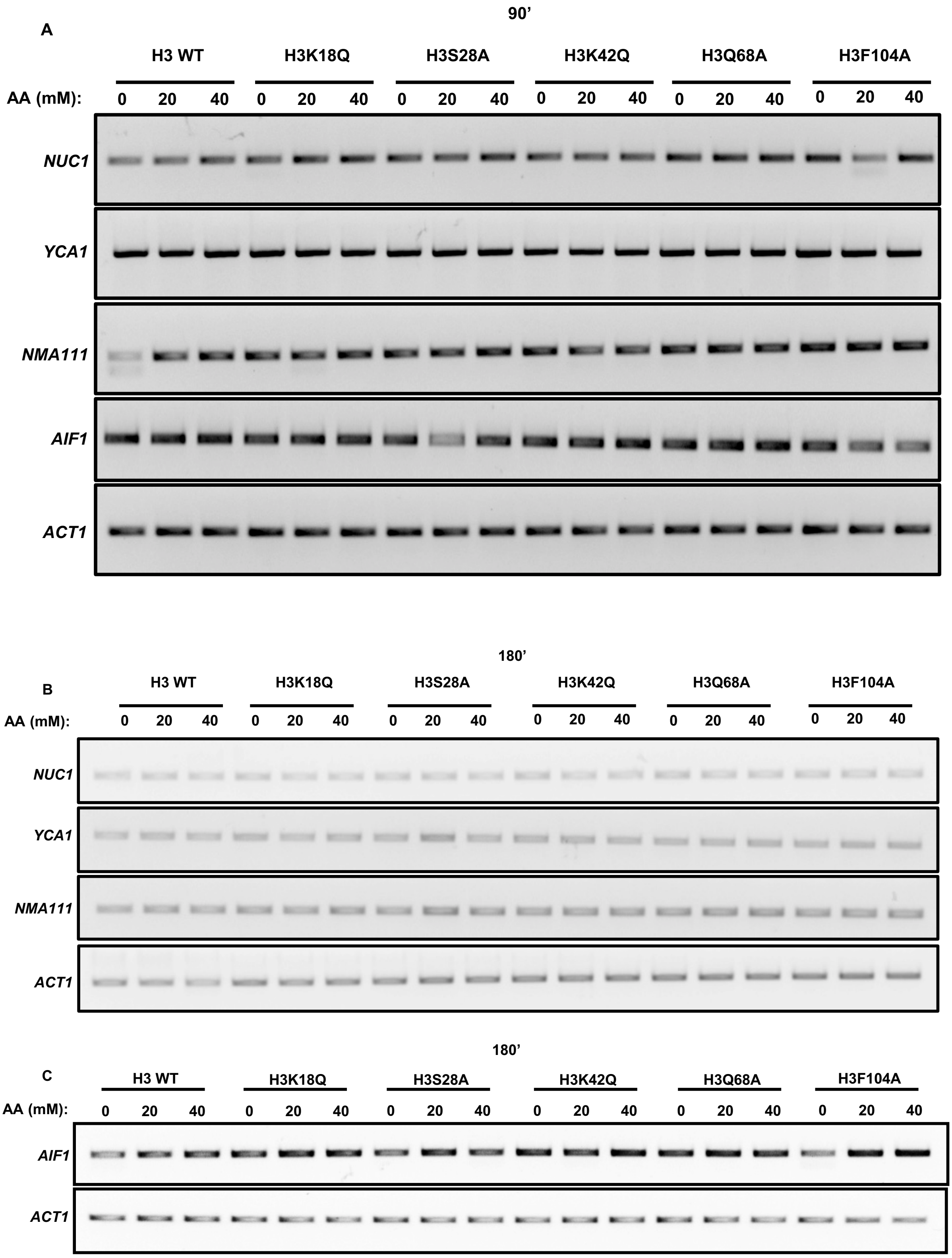

Figure S3. Expression of yeast apoptotic pathway genes in wild-type (H3 WT) and mutants. A, *YCA1*, *NMA111*, *AIF1* and *NUC1* gene levels were examined in cDNA prepared from wild-type (H3 WT) and acetic acid sensitive mutant cells under untreated/ acetic acid treated conditions (20, 40 mM) for 90' **B,** *NUC1*, *YCA1* and *NMA111* gene levels were examined in cDNA prepared from wild-type (H3 WT) and acetic acid sensitive mutant cells under untreated/ acetic acid treated conditions (20, 40 mM) for 180'. **C,** The *AIF1* gene level was examined in the same samples shown in B. *ACT1* expression serves as the control.

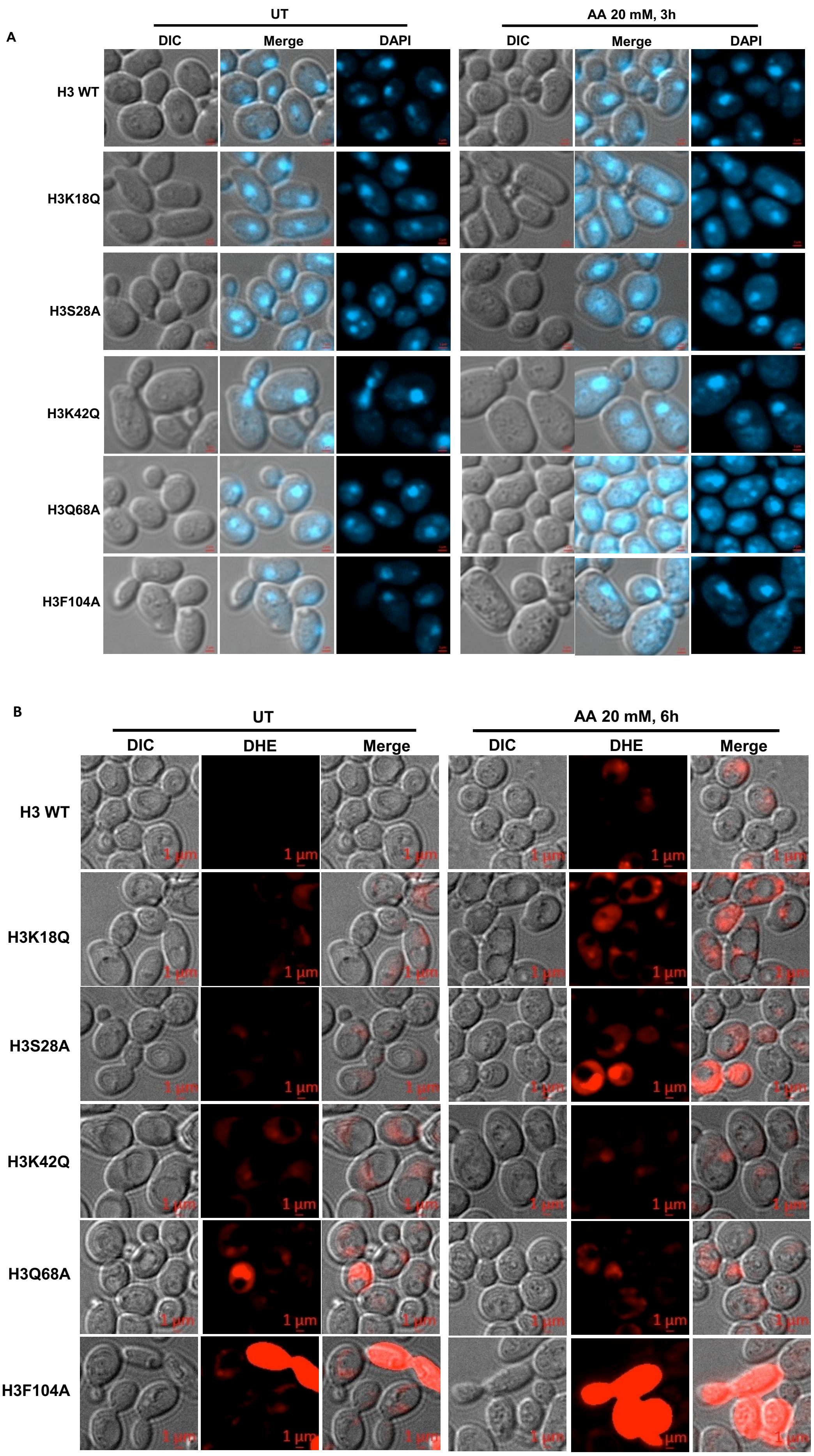
A
UT
AA 20 mM, 3h
DIC
Merge
DAPI
H3 WT
H3K18Q
H3S28A
H3K42Q
H3Q68A
H3F104A
B
UT
AA 20 mM, 6h
DIC
DHE
Merge
1 µm

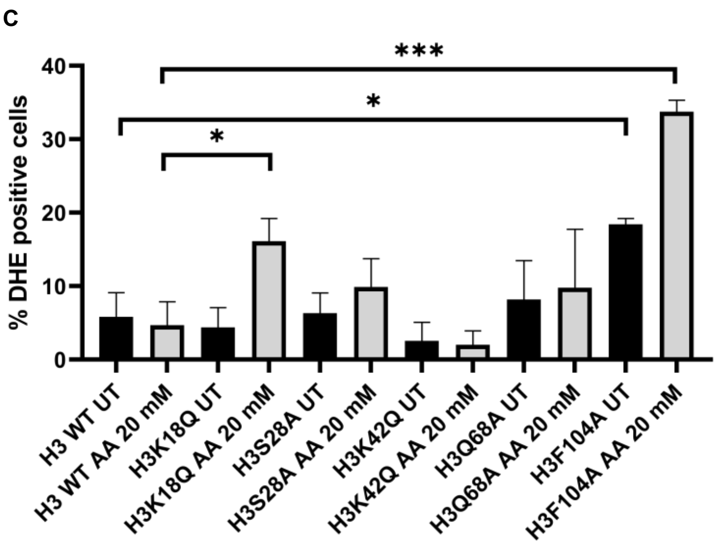

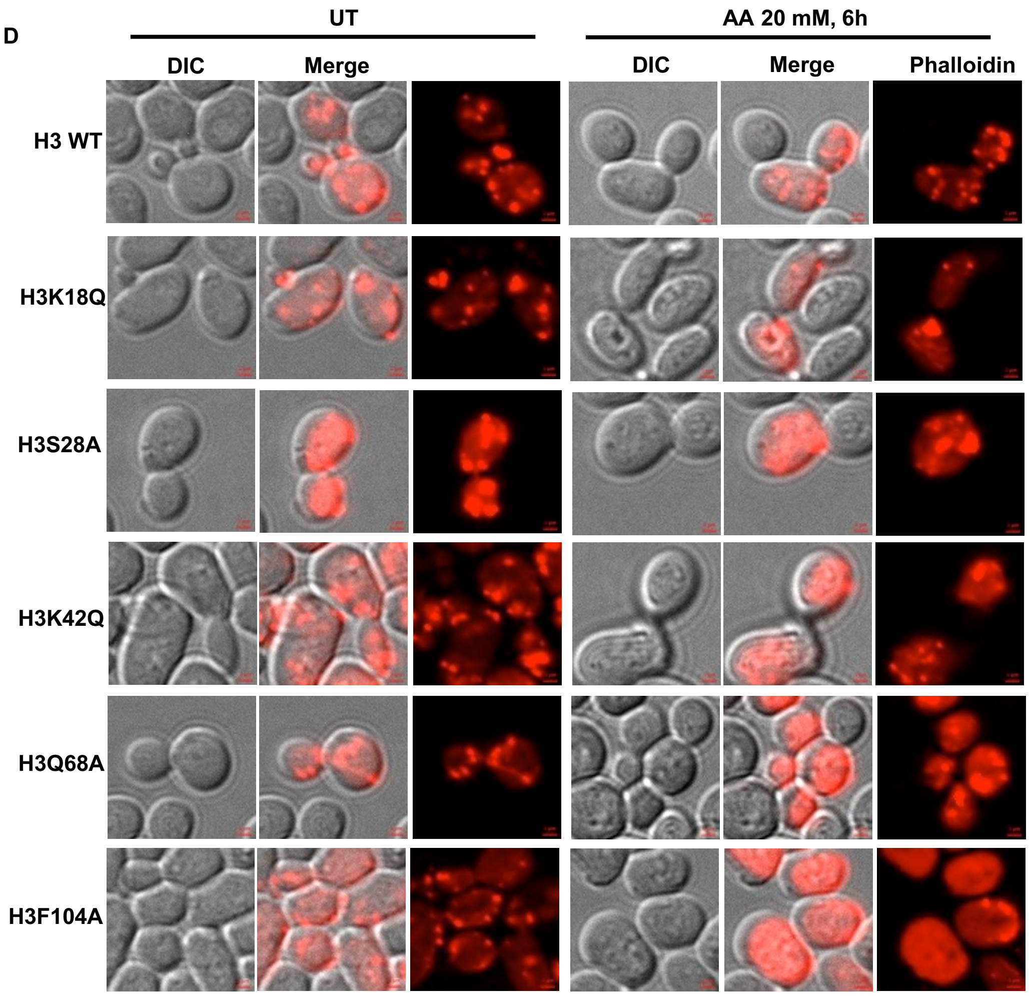

Figure S4. Acetic acid-sensitive mutants exhibit chromatin fragmentation, enhanced ROS generation and aggregated actin cytoskeleton after treatment with acetic acid. A, DAPI **B,** DHE (for ROS) **C,** Quantification of DHE+ cells. **D,** Phalloidin (for actin cytoskeleton) staining images of wild-type (H3 WT) and the mutant cells, which were grown to an absorbance of ~1 at 600 nm and then left untreated (UT) or treated with acetic acid for the indicated time and concentration. ∗∗∗p < 0.001, ∗∗p (0.001 to 0.01), ∗p (0.01 to 0.05).

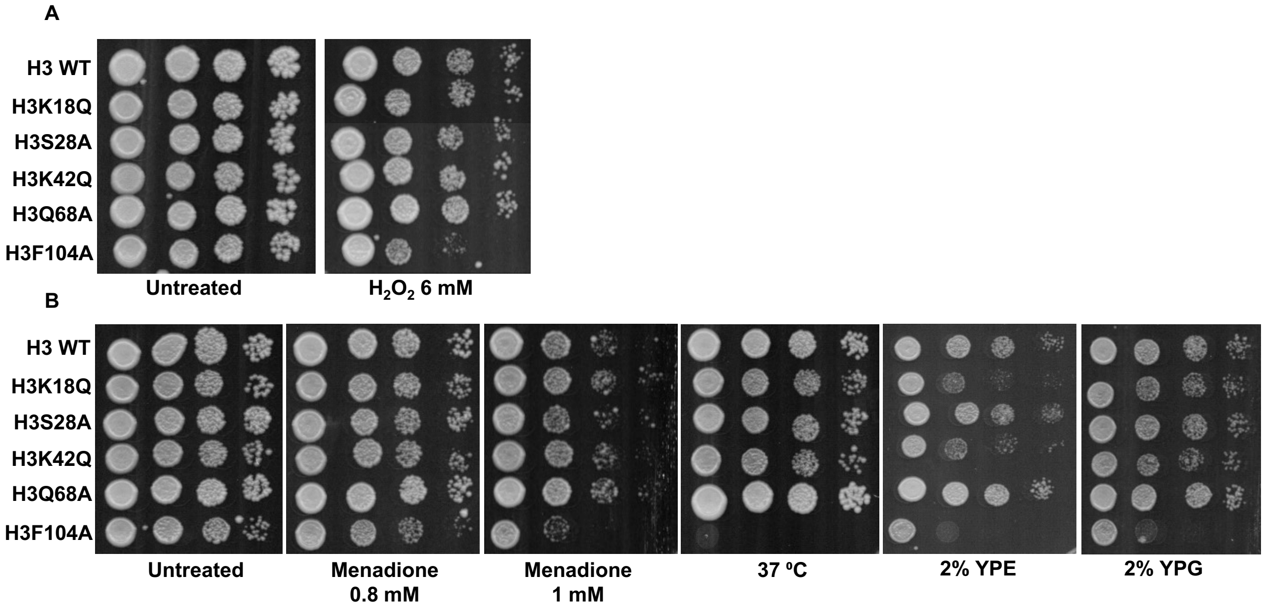

Figure S5. Tolerance of Histone H3 point mutants against oxidative stress and non-fermentable carbon source. A, Spot-test assays of the wild-type (H3 WT) and acetic acid sensitive mutants in the presence of different stress conditions H_2O_2 (6 mM) or **B,** menadione (0.8 mM and 1 mM), YPE (yeast extract, peptone and 2% ethanol), YPG (yeast extract, peptone and 2% glycerol) or 37 ^{o}C. Plates were incubated at 30 °C for 72 h and scanned. Please note that the plates of YPE/YPG were incubated for 120 h before scanning, and the untreated plate data at 48h has been shown as the spots become completely saturated at 120 h.

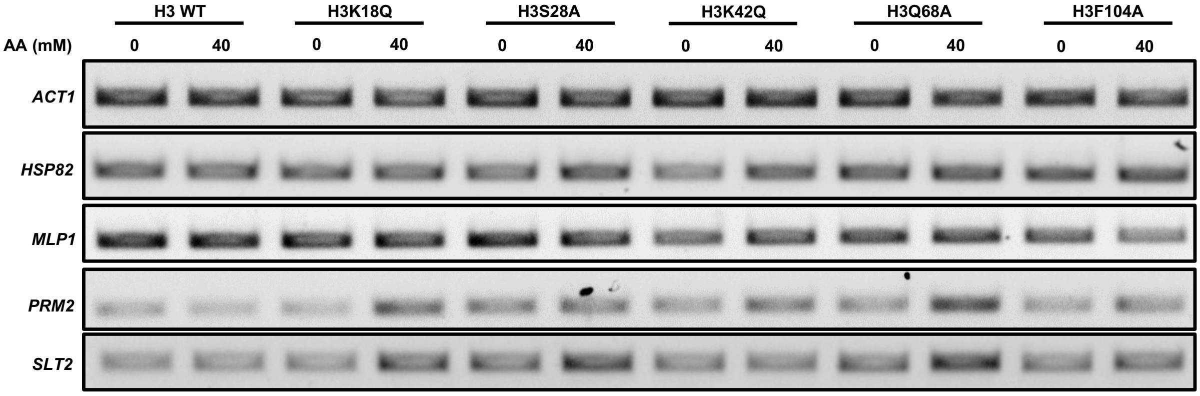

Figure S6. Expression of CWI pathway genes in acetic acid sensitive mutants. *MLP1*, *PRM2, PRM1*, *SLT2* and *HSP82* level was examined in cDNA prepared from H3 WT and indicated mutants untreated or treated with acetic acid (40-mM) for 6h. *ACT1* gene expression was used as the control.

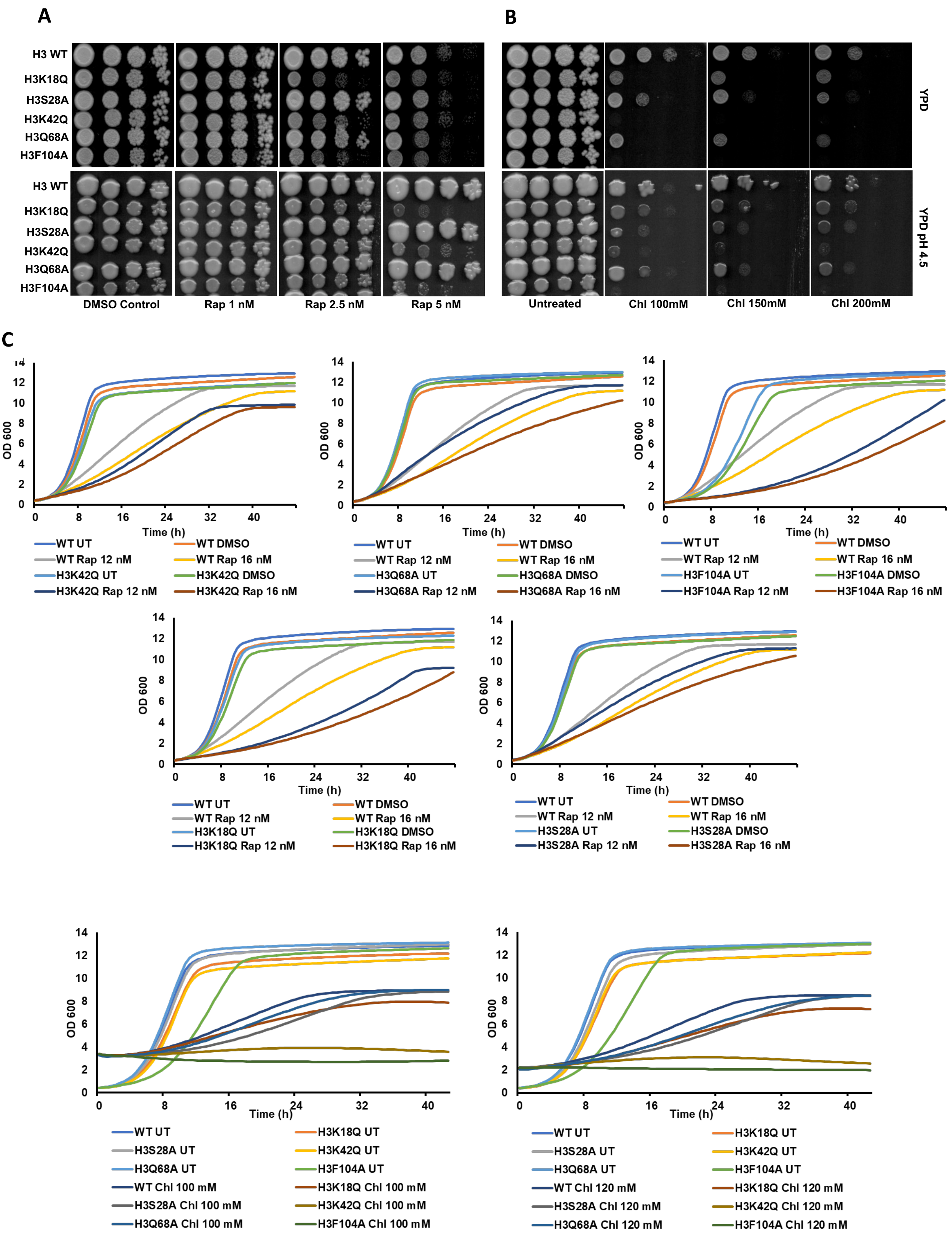

Figure S7. Tolerance of acetic acid-sensitive mutants against chloroquine (Chl) or rapamycin (Rap). A, Spot-test assays of the wild-type (H3 WT) and acetic acid sensitive mutants in the presence of Rap (1, 2.5, 5 nM) or **B,** Chl concentrations (100, 150, 200 mM). Plates were incubated at 30 °C for 72 h (Tm) or 96h (Rap, Chl) and photographed. **C**, Growth curves of the indicated strains in liquid culture under untreated (UT) or rapamycin (Rap) (12, 16 nM) or chloroquine (Chl 100, 120 mM) conditions. The graphs reflect the absorbance values at 600 nm at every 30' for the hours indicated in the x-axis.

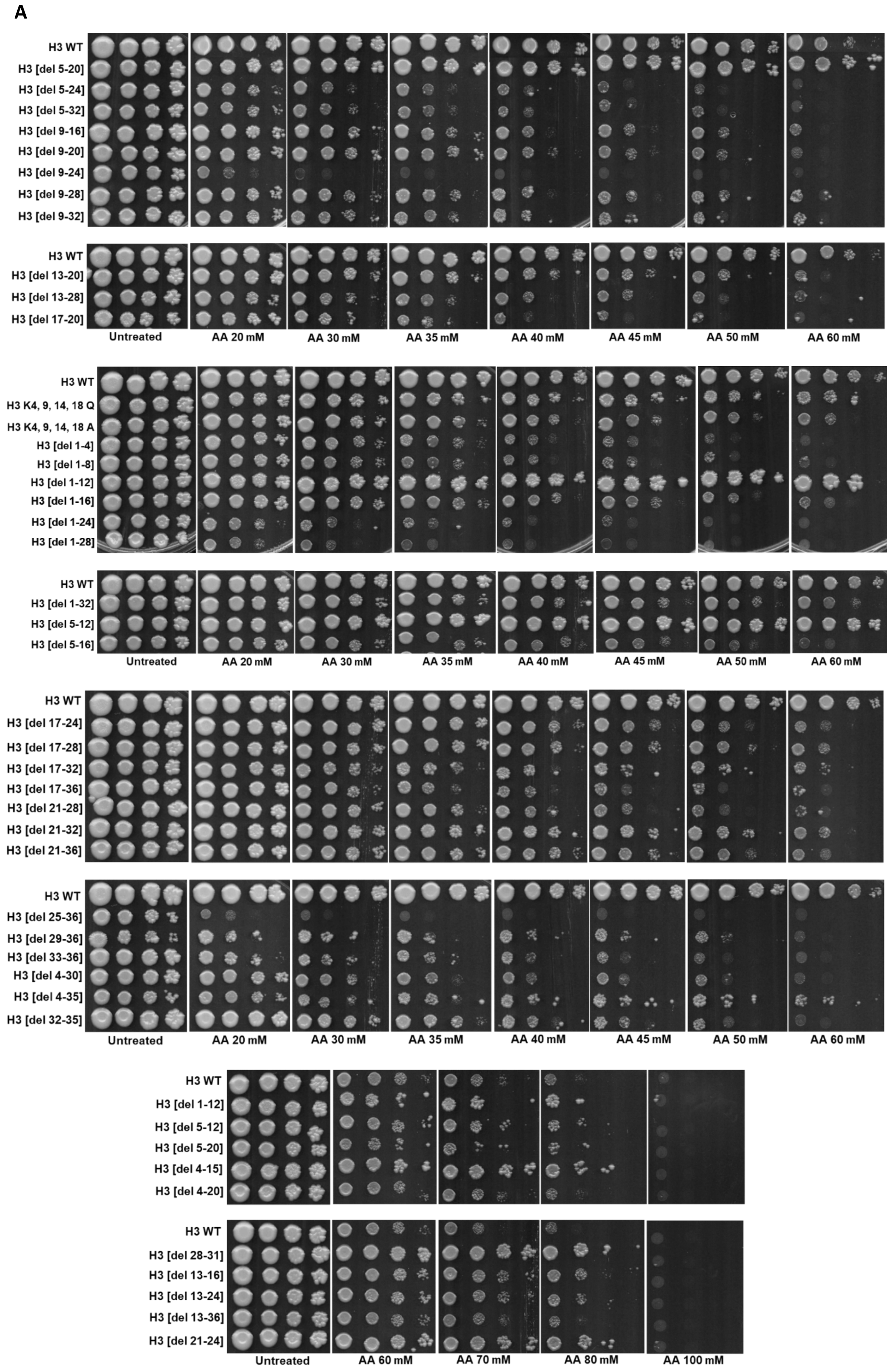
A
H3 WT
H3 [del 5-20]
H3 [del 5-24]
H3 [del 5-32]
H3 [del 9-16]
H3 [del 9-20]
H3 [del 9-24]
H3 [del 9-28]
H3 [del 9-32]
H3 WT
H3 [del 13-20]
H3 [del 13-28]
H3 [del 17-20]
Untreated
AA 20 mM
AA 30 mM
AA 35 mM
AA 40 mM
AA 45 mM
AA 50 mM
AA 60 mM
H3 WT
H3 K4, 9, 14, 18 Q
H3 K4, 9, 14, 18 A
H3 [del 1-4]
H3 [del 1-8]
H3 [del 1-12]
H3 [del 1-16]
H3 [del 1-24]
H3 [del 1-28]
H3 WT
H3 [del 1-32]
H3 [del 5-12]
H3 [del 5-16]
Untreated
AA 20 mM
AA 30 mM
AA 35 mM
AA 40 mM
AA 45 mM
AA 50 mM
AA 60 mM
H3 WT
H3 [del 17-24]
H3 [del 17-28]
H3 [del 17-32]
H3 [del 17-36]
H3 [del 21-28]
H3 [del 21-32]
H3 [del 21-36]
H3 WT
H3 [del 25-36]
H3 [del 29-36]
H3 [del 33-36]
H3 [del 4-30]
H3 [del 4-35]
H3 [del 32-35]
Untreated
AA 20 mM
AA 30 mM
AA 35 mM
AA 40 mM
AA 45 mM
AA 50 mM
AA 60 mM
H3 WT
H3 [del 1-12]
H3 [del 5-12]
H3 [del 5-20]
H3 [del 4-15]
H3 [del 4-20]
H3 WT
H3 [del 28-31]
H3 [del 13-16]
H3 [del 13-24]
H3 [del 13-36]
H3 [del 21-24]
Untreated
AA 60 mM
AA 70 mM
AA 80 mM
AA 100 mM

B

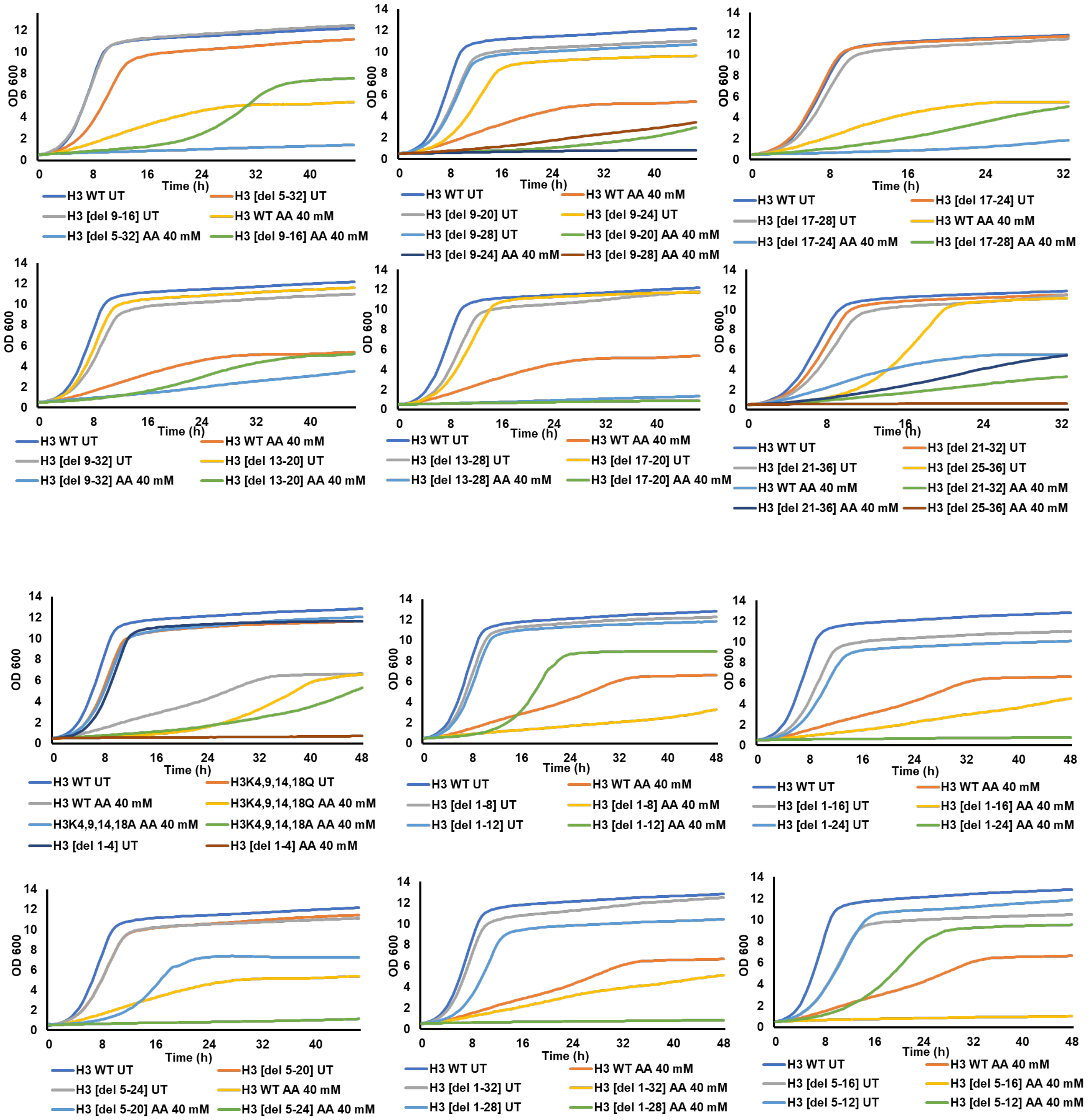
OD 600
Time (h)
H3 WT UT
H3 [del 5-32] UT
H3 [del 9-16] UT
H3 WT AA 40 mM
H3 [del 5-32] AA 40 mM
H3 [del 9-16] AA 40 mM
OD 600
Time (h)
H3 WT UT
H3 WT AA 40 mM
H3 [del 9-20] UT
H3 [del 9-24] UT
H3 [del 9-28] UT
H3 [del 9-20] AA 40 mM
H3 [del 9-24] AA 40 mM
H3 [del 9-28] AA 40 mM
OD 600
Time (h)
H3 WT UT
H3 [del 17-24] UT
H3 [del 17-28] UT
H3 WT AA 40 mM
H3 [del 17-24] AA 40 mM
H3 [del 17-28] AA 40 mM
OD 600
Time (h)
H3 WT UT
H3 WT AA 40 mM
H3 [del 9-32] UT
H3 [del 13-20] UT
H3 [del 9-32] AA 40 mM
H3 [del 13-20] AA 40 mM
OD 600
Time (h)
H3 WT UT
H3 WT AA 40 mM
H3 [del 13-28] UT
H3 [del 17-20] UT
H3 [del 13-28] AA 40 mM
H3 [del 17-20] AA 40 mM
OD 600
Time (h)
H3 WT UT
H3 [del 21-32] UT
H3 [del 21-36] UT
H3 [del 25-36] UT
H3 WT AA 40 mM
H3 [del 21-32] AA 40 mM
H3 [del 21-36] AA 40 mM
H3 [del 25-36] AA 40 mM
OD 600
Time (h)
H3 WT UT
H3K4,9,14,18Q UT
H3 WT AA 40 mM
H3K4,9,14,18Q AA 40 mM
H3K4,9,14,18A AA 40 mM
H3K4,9,14,18A AA 40 mM
H3 [del 1-4] UT
H3 [del 1-4] AA 40 mM
OD 600
Time (h)
H3 WT UT
H3 WT AA 40 mM
H3 [del 1-8] UT
H3 [del 1-8] AA 40 mM
H3 [del 1-12] UT
H3 [del 1-12] AA 40 mM
OD 600
Time (h)
H3 WT UT
H3 WT AA 40 mM
H3 [del 1-16] UT
H3 [del 1-16] AA 40 mM
H3 [del 1-24] UT
H3 [del 1-24] AA 40 mM
OD 600
Time (h)
H3 WT UT
H3 [del 5-20] UT
H3 [del 5-24] UT
H3 WT AA 40 mM
H3 [del 5-20] AA 40 mM
H3 [del 5-24] AA 40 mM
OD 600
Time (h)
H3 WT UT
H3 WT AA 40 mM
H3 [del 1-32] UT
H3 [del 1-32] AA 40 mM
H3 [del 1-28] UT
H3 [del 1-28] AA 40 mM
OD 600
Time (h)
H3 WT UT
H3 WT AA 40 mM
H3 [del 5-16] UT
H3 [del 5-16] AA 40 mM
H3 [del 5-12] UT
H3 [del 5-12] AA 40 mM

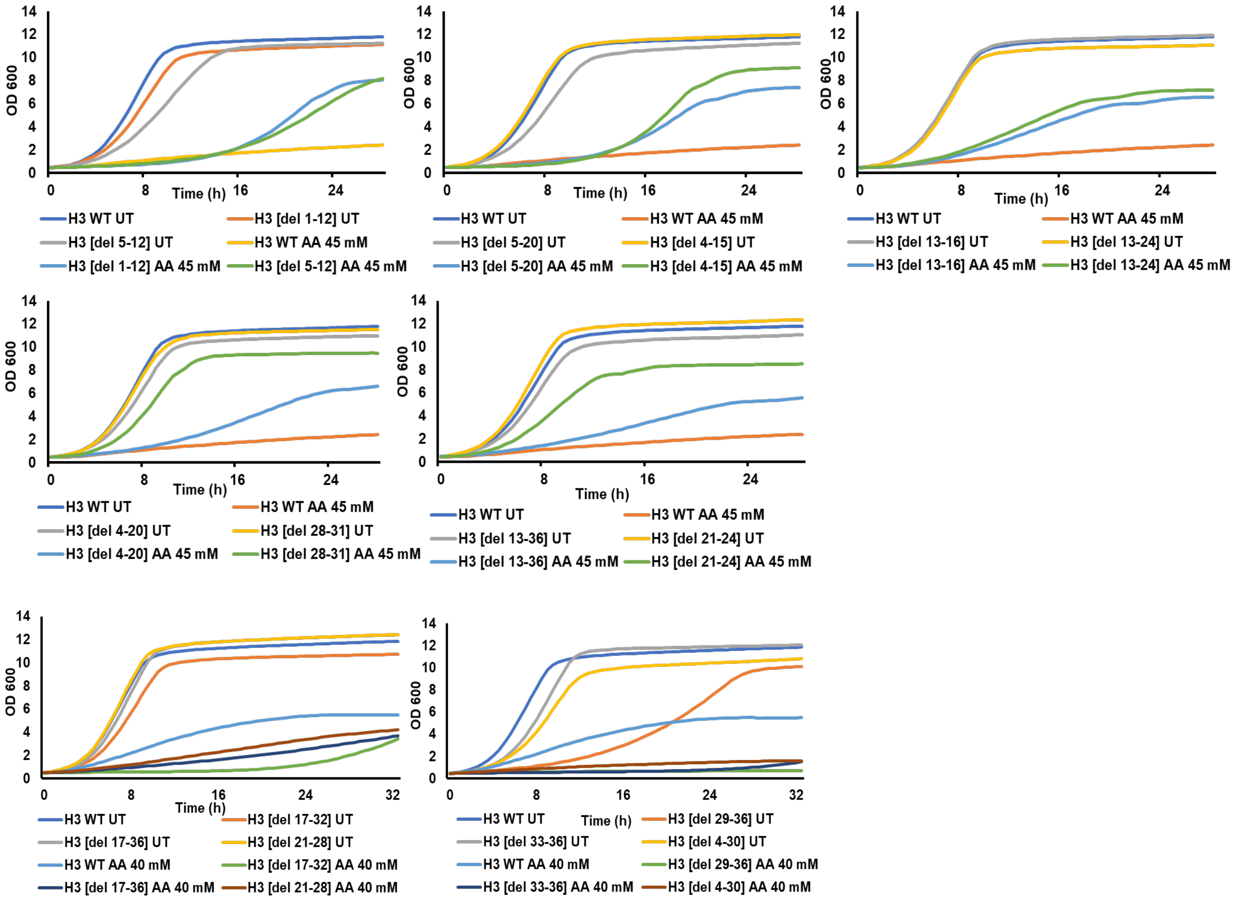

Figure S8. Screening of Histone H3 N-terminal tail truncation mutants in acetic acid. A, Spot-test assays of the wild-type (H3 WT) and H3 N-terminal tail truncation mutants in different acetic acid concentrations (20, 30, 35, 40, 45, 50, 60, 70, 80, 100 mM). Higher concentrations of acetic acid are used only for resistant strains identified in the primary screening. Plates were incubated at 30 °C for 72 h and scanned. **B,** Growth of the H3 WT and N-terminal tail truncation mutant cells in liquid culture under untreated (UT) or acetic acid treated (AA) conditions. The graphs reflect the absorbance values at 600 nm at every 30’ for the hours indicated in the x-axis.

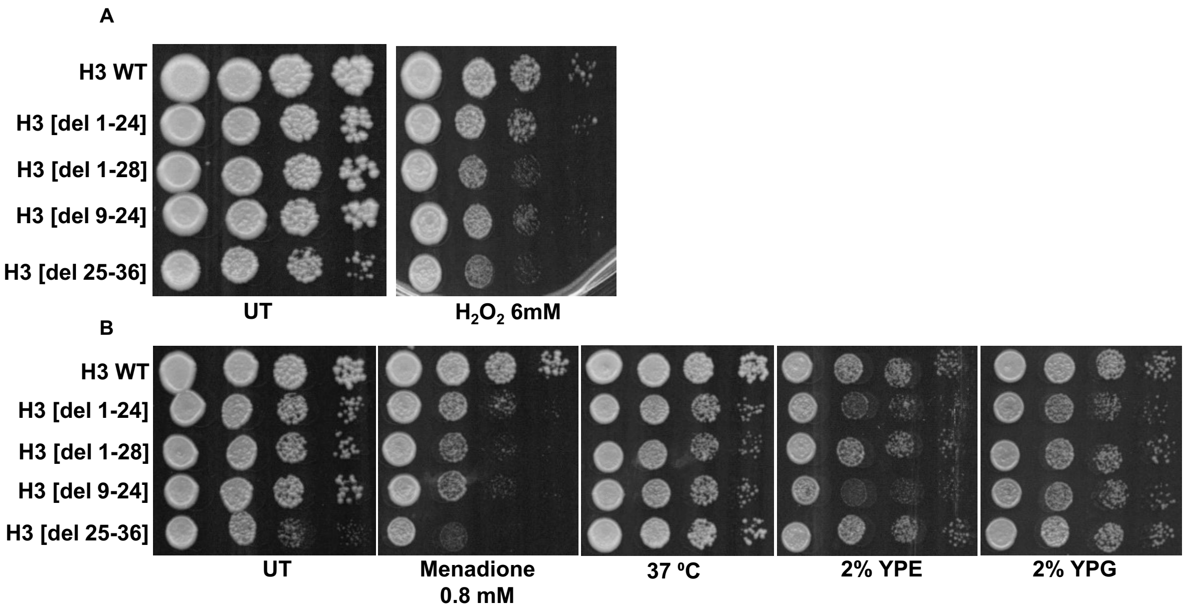

Figure S9. Tolerance of N-terminal tail truncations against oxidative stress and non-fermentable carbon source. A, Spot-test assays of the wild-type (H3 WT) and acetic acid sensitive mutants in the presence of different stress conditions H_2O_2 (6 mM), **B,** menadione (0.8 mM and 1 mM), YPE (yeast extract, peptone and 2% ethanol), YPG (yeast extract, peptone and 2% glycerol) or 37 ºC. Plates were incubated at 30 °C for 72 h and scanned. Please note that the plates of YPE/YPG were incubated for 120 h before scanning, and the untreated plate data at 48h has been shown as the spots become completely saturated at 120 h.

Table S1: List of histone H3 mutants used in the study.

All strains are isogenic to *MATa his3Δ200 leu2Δ0 lys2Δ0 trp1Δ63 ura3Δ0 met15Δ0 can1::MFA1pr-HIS3 hht1-hhf1::NatMX4 hht2-hhf2::[HHTS-HHFS]*-URA3*

1	H3 WT	25	H3 [del 13-24]	49	H3K4,9,14,18Q
2	H3K18Q	26	H3 [del 13-28]	50	H3K4,9,14,18A
3	H3S28A	27	H3 [del 13-32]	51	H3 [del 1-4]
4	H3K42Q	28	H3 [del 13-36]	52	H3 [del 1-8]
5	H3Q68A	29	H3 [del 17-20]	53	H3 [del 1-12]
6	H3F104A	30	H3 [del 17-24]	54	H3 [del 1-16]
7	H3K9R	31	H3 [del 17-28]	55	H3 [del 1-20]
8	H3K37A	32	H3 [del 17-32]	56	H3 [del 1-24]
9	H3Y41F	33	H3 [del 17-36]	57	H3 [del 1-28]
10	H3E105A	34	H3 [del 21-24]	58	H3 [del 1-32]
11	H3R134A	35	H3 [del 21-28]	59	H3 [del 9-32]
12	H3R49A	36	H3 [del 21-32]	60	H3 [del 9-36]
13	H3R63A	37	H3 [del 21-36]	61	H3 [del 13-16]
14	H3R69K	38	H3 [del 25-28]	62	H3 [del 13-20]
15	H3189A	39	H3 [del 25-32]	63	H3 [del 32-35]
16	H3 [del 5-8]	40	H3 [del 25-36]	64	H3 [del 9-28]
17	H3 [del 5-12]	41	H3 [del 29-32]	65	H3 [del 4-20]
18	H3 [del 5-16]	42	H3 [del 29-36]	66	H3 [del 4-30]
19	H3 [del 5-20]	43	H3 [del 33-36]	67	H3 [del 4-35]
20	H3 [del 5-24]	44	H3 [del 1-20]	68	H3 [del 28-31]
21	H3 [del 5-28]	45	H3 [del 1-28]	69	H3 *aif1Δ*
22	H3 [del 5-32]	46	H3 [del 4-15]	70	H3S28A *aif1Δ*
23	H3 [del 9-20]	47	H3 [del 9-24]	71	H3Q68A *aif1Δ*
24	H3 [del 9-12]	48	H3 [del 9-16]		

Table S2: List of primers used in this study.

Primers Name	Primers Sequence (5'-3')	References
ACT1-F	TCGTCGGTAGACCAAGACAC	This study
ACT1-R	TTCTTCTGGGGCAACTCTCA	This study
AIF1-F	GCTGTCCGTTTGACGGTTTC	This study
AIF1-R	GATCAGCCCACTTTGAGCCA	This study
YCA1-F	TCCTCCACCTAACCAGCAGT	This study
YCA1-R	TTGTGCCTTTGCCTGTTCCT	This study
NMA111-F	AGTTTGGCTAAGGTCGGCTC	This study
NMA111-R	AACCACTTGAACCGCCAGAA	This study
NUC1-F	TGCAGAACCGCGAAGAGTTT	This study
NUC1-R	CCTCGATCATAGCCCGACCT	This study
SLT2-F	ACCTGCCACTGGAAATACCG	This study
SLT2-R	AGAAGTGGCGCGAATTCACTA	This study
RLM1-F	GCTGCCACCGCATATAATGG	This study
RLM1-R	TATCCCTAGCGGATCTGCCG	This study
HSP82-F	AGAGCAACGACGACGAACAA	This study
HSP82-R	ATTGGATTGGGTAGGCCACG	This study
MLP1-F	ACATTGCGGAATGAGCTGGA	This study
MLP1-R	TGTGAGCGCTTTTTCCAACG	This study
PRM2-F	GAGCGGGACCTTCGCTATAC	This study
PRM2-R	GGATGTCCGTTGATTGCACTT	This study
PRM5-F	TGAAAAGGAGGTGGGTGAAGAC	This study
PRM5-R	CATCCTGTTCTTTCCCCTCCAG	This study
AIF1KO-F	AGGAAAGAGCAGAGAAAGGAAGAAAGAAATTGCAAAATAT CTGTGCGGTATTTCACACCG	This study
AIF1KO-R	ATATATATATATATATATACGCTGCAGTTCATATTTTAGTAGATTGTACTGAGAGTGCAC	This study
